# *O*-GlcNAcylation Suppresses the Ion Current IClswell by Preventing the Binding of the Protein ICln to α-Integrin

**DOI:** 10.3389/fcell.2020.607080

**Published:** 2020-11-19

**Authors:** Roberta Costa, Alessia Remigante, Davide A. Civello, Emanuele Bernardinelli, Zoltán Szabó, Rossana Morabito, Angela Marino, Antonio Sarikas, Wolfgang Patsch, Markus Paulmichl, Tamás Janáky, Attila Miseta, Tamás Nagy, Silvia Dossena

**Affiliations:** ^1^Institute of Pharmacology and Toxicology, Paracelsus Medical University, Salzburg, Austria; ^2^Department of Chemical, Biological, Pharmaceutical and Environmental Sciences, University of Messina, Messina, Italy; ^3^Department of Medical Chemistry, Faculty of Medicine, University of Szeged, Szeged, Hungary; ^4^Department of Personalized Medicine, Humanomed, Klagenfurt, Austria; ^5^Department of Laboratory Medicine, Medical School, University of Pécs, Pécs, Hungary

**Keywords:** *O*-GlcNAcylation, IClswell, ICln, α-integrin, regulatory volume decrease, kidney, protein–protein interaction

## Abstract

*O*-GlcNAcylation is a post-translational modification of proteins that controls a variety of cellular processes, is chronically elevated in diabetes mellitus, and may contribute to the progression of diabetic complications, including diabetic nephropathy. Our previous work showed that increases in the *O*-GlcNAcylation of cellular proteins impair the homeostatic reaction of the regulatory volume decrease (RVD) after cell swelling by an unknown mechanism. The activation of the swelling-induced chloride current IClswell is a key step in RVD, and ICln, a ubiquitous protein involved in the activation of IClswell, is *O*-GlcNAcylated. Here, we show that experimentally increased *O*-GlcNAcylation of cellular proteins inhibited the endogenous as well as the ICln-induced IClswell current and prevented RVD in a human renal cell line, while decreases in *O*-GlcNAcylation augmented the current magnitude. In parallel, increases or decreases in *O*-GlcNAcylation, respectively, weakened or stabilized the binding of ICln to the intracellular domain of α-integrin, a process that is essential for the activation of IClswell. Mutation of the putative YinOYang site at Ser67 rendered the ICln-induced IClswell current unresponsive to *O*-GlcNAc variations, and the ICln interaction with α-integrin insensitive to *O*-GlcNAcylation. In addition, exposure of cells to a hypotonic solution reduced the *O*-GlcNAcylation of cellular proteins. Together, these findings show that *O*-GlcNAcylation affects RVD by influencing IClswell and further indicate that hypotonicity may activate IClswell by reducing the *O*-GlcNAcylation of ICln at Ser67, therefore permitting its binding to α-integrin. We propose that disturbances in the regulation of cellular volume may contribute to disease in settings of chronically elevated *O*-GlcNAcylation, including diabetic nephropathy.

## Introduction

*O*-GlcNAcylation is a dynamic and abundant post-translational modification of cytosolic, nuclear, mitochondrial, and membrane proteins, which consists of the addition of a single monosaccharide (*O*-linked β-D-*N*-acetylglucosamine or *O*-GlcNAc) to hydroxyl groups of serine and/or threonine residues. The substrate for *O*-GlcNAcylation is uridine diphosphate-β-*N*-acetylglucosamine (UDP-GlcNAc), the end product of the hexosamine biosynthetic pathway (HBP), and its abundance controls the efficiency of protein *O*-GlcNAcylation. Approximately 2–5% of cellular glucose is diverted to the HBP, which is consequently strongly connected to the nutrient availability and carbohydrate metabolism (Wells et al., 2001, 2003b; Zachara and Hart, 2006; Zachara et al., 2015; Fisi et al., 2017). *O*-GlcNAc modification is reversible and specific and is tightly controlled by the activity of the enzymes *O*-GlcNAc-transferase (OGT) and *O*-GlcNAcase (OGA), which catalyze the addition and the removal of *O*-GlcNAc from target proteins, respectively (Gloster and Vocadlo, 2010; Yang and Qian, 2017).

*O*-GlcNAcylation may compete and establish a dynamic interplay with other post-translational modifications, including phosphorylation, by occurring on same sites or adjacent sites (Slawson and Hart, 2011). Amino acids that are either phosphorylated or *O*-GlcNAcylated in a mutually exclusive manner are called YinOYang sites and are crucially involved in controlling protein activity (Zeidan and Hart, 2010).

*O*-GlcNAcylation regulates a variety of cellular and molecular processes including intracellular signaling, gene transcription, protein–protein interaction, and stability, localization and expression of proteins (Butkinaree et al., 2010). Consequently, *O*-GlcNAc plays a role in numerous physiological and pathological conditions, including the response to stress, cancer, neurodegeneration, and diabetes mellitus (Wells et al., 2001; Zachara et al., 2004; Hart et al., 2007; Fardini et al., 2013). Specifically, strong positive correlations between protein *O*-GlcNAc levels and insulin resistance, glucose toxicity (McClain and Crook, 1996; Copeland et al., 2008), and chronic complications of diabetes mellitus (Brownlee, 2001; Buse, 2006; McLarty et al., 2013) have been well established in cell culture and animal models (Buse, 2006; Dias and Hart, 2007; Nagy et al., 2007) as well as in patients (Wang et al., 2009; Park et al., 2010; Springhorn et al., 2012; Myslicki et al., 2014; Pagesy et al., 2018). Among the multiple and diverse functions of *O*-GlcNAcylation, one of the least studied is the modulation of the regulation of cellular volume.

The regulation of cellular volume is a homeostatic reaction essential to normal cell function (Lang et al., 1998; Hoffmann et al., 2009). Variations of the cellular volume are due to an imbalance between the intra- and the extracellular osmolality; may induce alterations in cell morphology, function, and survival; and therefore require counterbalance. In mammalian cells, swelling activates a rapid release of KCl and/or non-essential organic osmolytes accompanied by cellular water (Jakab et al., 2002). This process is called regulatory volume decrease (RVD) and permits swollen cells to regain their original volume. The main conductive pathway leading to the release of Cl^–^ during RVD is called IClswell (Furst et al., 2002a), and the respective main molecular entity was identified as the leucine-rich repeat containing protein 8A (LRRC8A) (Qiu et al., 2014; Voss et al., 2014; Pedersen et al., 2016), although other channels may also play a role in this context (Liu et al., 2019; Okada et al., 2019).

Our previous study established a link between *O*-GlcNAcylation and RVD. Increasing the cellular *O*-GlcNAc levels by a hour pretreatment with glucosamine, PUGNAc, or high glucose (30 mM) altered the response of Jurkat cells to a hypotonic stimulus. Specifically, the swelling observed following exposure of cells to a hypotonic extracellular medium was augmented and the intracellular water diffusion was reduced (Nagy et al., 2010). However, the molecular mechanism by which augmented *O*-GlcNAcylation affects RVD remains to be established.

ICln is a ubiquitous, highly conserved, multifunctional protein that plays a fundamental role in RVD (Paulmichl et al., 1992; Furst et al., 2006). During a hypotonic stimulus, ICln is transposed toward the plasma membrane and facilitates the activation of IClswell (Ritter et al., 2003; Dossena et al., 2011). Binding of cytosolic ICln to the intracellular domain of α-integrin is an essential prerequisite for ICln association with the plasma membrane and IClswell activation and requires a direct molecular interaction between highly conserved amino acid motifs of both proteins (Schedlbauer et al., 2019). Specifically, the sequence (61)ISLHA(65) (single-letter amino acid code; numbers refer to the amino acid position within the human ICln protein sequence), located in the β4–β5 loop and β5 sheet of the pleckstrin homology-like N-terminal portion of ICln (Furst et al., 2005), appears to be essential for binding to α-integrin as well as for the ICln-induced activation of IClswell. Interestingly, ICln is *O*-GlcNAcylated (Hahne et al., 2013); however, the ICln *O*-GlcNAc modification site(s) as well as their physiological significance are not assessed.

The aim of the present study was to explore the mechanism by which *O*-GlcNAcylation affects RVD. Here, we show that augmented *O*-GlcNAcylation prevents RVD by reducing the magnitude of the ion current IClswell. IClswell inhibition results from *O*-GlcNAcylation of the protein ICln on serine 67, which prevents its binding to the intracellular domain of the integrin α chain. These findings suggest that dysfunction of cellular volume regulation may contribute to diseases associated with chronic elevation of *O*-GlcNAcylation.

## Materials and Methods

### Cell Culture and Transfection

Human embryonic kidney (HEK) 293 Phoenix (DiCiommo et al., 2004) and HeLa cells [human cervical adenocarcinoma, CCL-2, obtained from American Type Cell Culture Collection (ATCC), Manassas, VA, United States] were cultured in Minimum Essential Eagle Medium (Sigma-Aldrich, St. Louis, MO, United States) supplemented with 10% fetal bovine serum (GIBCO, Thermo Fisher, Waltham, MA, United States), 2 mM L-glutamine, 100 U/ml penicillin, 100 μg/ml streptomycin, and 1 mM pyruvic acid (sodium salt). NIH/3T3 cells (mouse embryonic fibroblasts, CRL-1658, obtained from ATCC) were cultured in Dulbecco’s Modified Eagle’s Medium (Sigma-Aldrich) supplemented with 10% bovine calf serum (GIBCO), 100 U/ml penicillin and 100 μg/ml streptomycin. The cells were maintained at 37°C, 5% CO_2_, 95% air, and 100% humidity. Subcultures were routinely established every second to third day by seeding the cells into 100-mm diameter Petri dishes following trypsin/ethylenediaminetetraacetic acid (EDTA) treatment.

For transfection, cells were seeded into six-well plates or 30-mm diameter Petri dishes, grown overnight to ∼50% confluence, and transiently transfected with 1–3 μg of plasmid DNA by the calcium phosphate co-precipitation method (HEK 293 Phoenix) or 1.5–3 μg of plasmid DNA and 3–6 μl METAFECTENE PRO^®^ (Biontex, Munich, Germany), following the manufacturer’s instructions (HeLa and NIH/3T3). For co-transfection experiments, equimolar amounts of two vectors were used. Medium was replaced 6–8 h after transfection.

### Plasmid Constructs

For electrophysiology experiments, the open reading frames (ORFs) coding for human ICln (hICln, NCBI Reference Sequence: NP_001284.1); *Canis familiaris* ICln (from Madin-Darby Canine Kidney cells, MDCK ICln, NP_001003288.1); and the long (OGA-L, NP_036347.1) or short (OGA-S, NP_001135906.1) isoforms of OGA, OGT (NP_858051.1; all from HEK 293 Phoenix cells), or integrin αIIbshort (amino acids 987–1039 of the human integrin αIIb chain, NP_000410.2) were cloned into the bicistronic mammalian expression vectors pIRES2EGFP or pIRES2dsRedExpress (Clontech, Mountain View, CA, United States). The use of a vector bearing the internal ribosome entry site (IRES) allows for the simultaneous expression of the protein of interest (either OGT, OGA, integrin αIIbshort, or ICln) and the transfection marker (either the enhanced green fluorescent protein EGFP or the red fluorescent protein dsRED) as two individual proteins from the same bicistronic mRNA, without the production of fusion proteins (Morgan et al., 1992). Control experiments were conducted in cells transfected with the pIRES2EGFP-EGFP vector, where an additional EGFP coding sequence was cloned into the pIRES2EGFP expression vector. Consequently, the control vector contained two EGFP coding sequences (one in the multiple cloning site and one downstream of the IRES sequence).

Plasmids encoding for ICln Ser67Ala, Thr223Ala, and Ser193^∗^ were obtained by site-directed mutagenesis of vectors bearing the wild-type ICln cDNA with the QuikChange Site-Directed Mutagenesis Kit (Agilent, Santa Clara, CA, United States). Mutagenesis primers were designed in-house (Gene Runner, version 5.0.993 Beta) and obtained from Microsynth AG (Balgach, Switzerland).

To produce fusion proteins for fluorescence resonance energetic transfer (FRET) experiments, hICln, MDCK ICln, hOGT, hOGA, or integrin αIIbshort ORFs were cloned into the pEYFPC1, pEYFPN1, pECFPC1, or pECFPN1 mammalian expression vectors (Clontech) in frame with the ORF of the enhanced yellow fluorescent protein (EYFP) or enhanced cyan fluorescent protein (ECFP).

All plasmid inserts were sequenced prior to their use in experiments (Microsynth AG).

### Manipulation of *O*-GlcNAcylation of Cellular Proteins

To increase the *O*-GlcNAcylation levels of cellular proteins, cells were incubated for 1 h in complete medium with 20 mM D-glucose, 5 mM glucosamine, 100 μM *O*-(2-acetamido-2-deoxy-D-glucopyranosylidene)amino *N*-phenyl carbamate (PUGNAc; Sigma Aldrich), 100 μM PUGNAc, and 5 mM glucosamine (Nagy et al., 2010) or were transfected for 24 h with OGT. To reduce the *O*-GlcNAcylation levels of proteins, cells were transfected for 24 h with OGA. PUGNAc was dissolved in 100% dimethyl sulfoxide (DMSO) and stored at −20°C. To obtain equal osmolarity of control incubation media, 20 mM mannitol and 5 mM D-glucose were used instead of 20 mM D-glucose and 5 mM glucosamine.

### Patch Clamp

Cells were transferred to glass coverslips (10-mm diameter) 8 h after transfection, and electrophysiology measurements were performed 24–32 h after transfection. Single cells expressing the transfection marker EGFP, dsRed, or both could be identified by fluorescence microscopy using FITC (excitation: 480/30x, dicroic: 505 dclp, and emission: 535/40 m) and TRITC (excitation: 540/25x, dicroic: 565 dclp, and emission: 605/55 m) filter sets. Selected cells were voltage-clamped using the whole-cell patch-clamp technique as previously described (Gandini et al., 2008; Dossena et al., 2011; Tamma et al., 2011; Morabito et al., 2017; Schedlbauer et al., 2019). The resistance of the glass pipettes was 3–8 MΩ when filled with the pipette solution (CsCl 125 mM, MgCl_2_ 5 mM, EGTA 11 mM, ATP Mg^2+^ salt 2 mM, HEPES 10 mM, pH 7.2 adjusted with CsOH, and 300 mOsm/kg H_2_O). Fast exchange of the isotonic bath solution (NaCl 125 mM, CaCl_2_ 2.5 mM, MgCl_2_ 2.5 mM, HEPES 10 mM, mannitol 40 mM, pH 7.4 adjusted with NaOH, and 300 mOsm/kg H_2_O) with a hypotonic bath solution (NaCl 125 mM, CaCl_2_ 2.5 mM, MgCl_2_ 2.5 mM, HEPES 10 mM, pH 7.4 adjusted with NaOH, and 260 mOsm/kg H_2_O) was obtained using a perfusion system with a flow rate of 5 ml/min and a bath volume of ∼300 μl. For data acquisition, an EPC10 USB amplifier (HEKA Elektronik, Lambrecht, Germany) controlled by a Macintosh computer running the Patch Master (v2x32, HEKA Elektronik) software was used. Access resistance was monitored throughout the recordings and never exceeded thrice the recording electrode resistance. Fast and slow capacitance were compensated. All current measurements were filtered at 2.9 kHz and digitized at 50 kHz. To monitor the activation of the swelling-activated chloride current (IClswell), cells were held at 0 mV and step pulses of 400-ms duration were applied from 0 to 40 mV every 20 s. To establish the current to voltage (IV) relationship, step pulses of 500-ms duration were applied every 5 or 10 min from −120 to +100 mV in 20-mV increments from a holding potential of 0 mV. For data analysis, Fit Master (v2x32, HEKA Elektronik) and Excel (Microsoft, United States) software were used. The current values (pA) were normalized to the membrane capacitance (pF) to obtain the current density (pA/pF). Each data set was obtained from cells from at least three independent subcultures with control experiments in cells of the same subculture.

### Western Blotting

Cells were washed twice with ice-cold phosphate-buffered saline (PBS) and lysed on ice in a buffer containing 20 mM Tris-HCl, 150 mM NaCl, 1 mM EDTA, 0.1% NP40, and protease inhibitor cocktail (Roche, Basel, Switzerland). Subsequently, cellular debris was discarded by centrifugation at 16,000 × *g* for 30 min at 4°C and the supernatant was collected. Typically, 10 μg of total proteins were separated by a 7.5% polyacrylamide gel containing 0.1% SDS and electroblotted using a polyvinylidene fluoride membrane (Bio-Rad, Basel, Switzerland) by applying a constant voltage (75 V) for 2 h at 4°C. Membranes were blocked for 1 h at room temperature in 5% bovine serum albumin (BSA) diluted in Tris-buffered saline (150 mM NaCl and 15 mM Tris-HCl) containing 0.1% Tween 20 (TBST) and incubated overnight at 4°C with the following primary antibodies: mouse monoclonal IgM CTD110.6 (Cell Signaling Technology, Inc., Danvers, MA, United States), highly specific for *O*-glycosylated proteins (Kneass and Marchase, 2004), diluted 1:1,000, or rabbit polyclonal IgG against amino acids 151–164 of CLNS1A (Sigma Aldrich), diluted 1:2,000. The housekeeping proteins GAPDH or β-actin were detected with a goat (GenScript, Piscataway, NJ, United States) or rabbit (Cell Signaling) polyclonal antibody, respectively, both diluted 1:1,000. Membranes were washed three times in TBST and incubated for 1 h at room temperature with the secondary antibodies diluted 1:10,000 in PBS and 5% BSA (goat anti-mouse IgM, IRD-680 RD, donkey anti-goat, IRD-800 CW, or goat anti-rabbit, IRD-800 CW, all from LI-COR, Lincoln, NE, United States). The signal of immunocomplexes was revealed using the Odyssey (LI-COR) infrared imaging system. Densitometric analysis was done using the ImageJ (1.49v) software.

### Acceptor Photobleaching Fluorescence Resonance Energy Transfer

Cells were transferred to round 3-cm diameter glass slides 32 h post-transfection and fixed for 30 min with 4% paraformaldehyde in Hanks’ Balanced Salt Solution (HBSS) 48 h post-transfection. Imaging was performed in HBSS at room temperature. The FRET acceptor EYFP was excited with the 514-nm line of the Argon laser, and the emission was detected in the 525–600-nm range. The FRET donor ECFP was excited with the 405-nm laser, and the emission was detected in the 450–490-nm range. Imaging was performed by sequential acquisition with FRET AB-Wizard with a Leica TCS SP5II AOBS confocal microscope (Leica Microsystems, Wetzlar, Germany) equipped with a HCX PL APO 63x/1.20 Lambda blue water immersion objective and controlled by the LAS AF software (version 2.7.3.9723, Leica Microsystems). EYFP photobleaching in whole cells was obtained with 15 sequential illuminations at 514 nm (zoom factor 8×). FRET efficiency was calculated using the following formula:

(1) FRETefficiency=1-ECFPpre/ECFPpost

ECFPpre and ECFPpost refer to the ECFP intensity before and after EYFP photobleaching, respectively, and were determined either in the bleaching regions of interest (ROI) or in plasma membrane ROIs, as indicated. In the latter case, ROIs were drawn within the bleaching ROI, on the pre-bleaching EYFP image, in areas with a clear targeting of αIIbs-EYFP at the cell periphery.

### Cell Volume Measurements

Cell volume measurements were performed as previously described (Morabito et al., 2013, 2014, 2017). Briefly, HEK 293 Phoenix cells were transferred to round 3-cm diameter glass slides and successively placed in a perfusion chamber. Cell volume measurements were performed on cells sequentially exposed to the following: isotonic solution for 1 min and hypotonic solution (∼15% reduction of osmolality by omission of mannitol) for 2–3 min. Cell volume measurements were performed on roundish cells. For each experiment, 100–150 images/cell (1 image/1,293 s) were taken with a phase contrast microscope (Leica DMI 6000, with a HCX PL APO 63x/1.20 Lambda blue water immersion objective; Leica Microsystems). Cell diameter was measured for each image and, assuming the cell as a sphere, cell volume was calculated and expressed as *V*/*V*_0_, where *V* and *V*_0_ represent the volume of a cell at a given time and the average volume of the same cell in isotonic solution, respectively.

### Immunoprecipitation and Mass Spectrometry

For immunoprecipitation, cells were lysed on ice for 30 min in IP buffer (25 mM Tris-HCl, pH 7.4, 150 mM NaCl, 1 mM EDTA, 1% Triton X-100, 5% glycerol, 0.05% Na-azide, protease inhibitor cocktail, and 50 μM of PUGNAc). Next, the samples were sonicated for 5 s at 30% power (Bandelin Sonopuls). Finally, the samples were centrifuged at 4°C for 10 min at 3,000 rpm, and the supernatants were incubated with anti-ICln antibodies #1 or #2 at 4°C overnight on a rotary shaker. Anti-ICln #1 is a polyclonal antibody raised in rabbits against amino acids 214–237 of human ICln, while anti-ICln #2 (Santa Cruz Biotechnology, Cat. No.: SC-393525) is a mouse monoclonal antibody. Protein A-Sepharose beads (Sigma-Aldrich) were prepared as follows: beads were washed in 800 μl IP buffer and incubated for 30 min in 3% BSA/IP buffer at 4°C on a shaker to block non-specific binding sites. Next, immunoprecipitates were incubated with the Protein A beads on a rotary shaker at 4°C for 3 h. Next, the beads were washed in 1 ml of IP buffer twice, the last wash buffer was removed carefully, and 50 μl of elution buffer (0.1 M glycine, pH 2.8) was added to the beads and incubated for 10 min at room temperature. The eluate (IP) was kept and 6 μl of 1.5 M Tris-HCl (pH 8.8) was added to neutralize the pH. During the process, lysates including the antibodies (input) and flow-through (FT) from the beads were collected. All of the collected samples (input, FT, and IP) were adjusted with 4X sample buffer and processed on 7.5% polyacrylamide gel electrophoresis. The proteins were either transferred to PVDF membranes for western blot analysis or the gel was directly stained with Coomassie Brilliant Blue R-250 for further mass spectrometry analysis.

For mass spectrometry (MS), the protein bands of interest were cut out from the Coomassie-stained gels and digested by trypsin using the in-gel digestion protocol as described (Szabo et al., 2012). Briefly, excised gel bands were cut into small pieces, destained in 25 mM NH_4_HCO_3_ and 50% acetonitrile, dehydrated with acetonitrile, and dried. The gel pieces were rehydrated in 12.5 ng/μl trypsin (Promega, Madison, WI, United States) solution (in 25 mM NH_4_HCO_3_) and incubated overnight at 37°C. Peptides were extracted with 5% formic acid and twice with 60% acetonitrile in 1% formic acid.

Digested protein samples were analyzed using a Waters nanoACQUITY UPLC System coupled with a Thermo Q Exactive plus mass spectrometer. Forty-five-minute long gradients from 3% to 40% acetonitrile/water were applied on a Waters BEH130 C18 75 μm × 100 mm column with 1.7 μm particle size C18 packing. The mass spectrometer was operated in DDA mode, and the 12 most intensive peptides were selected for fragmentation in each MS scan.

From all acquired data, peaklists were generated by the Msconvert tool from ProteoWizard (Chambers et al., 2012). All MS/MS samples were analyzed using Mascot 2.2.06 (Matrix Science, London, UK). Mascot was set up to search the UniProt Human proteome database (2017.03 release, 70,942 entries) assuming digestion by trypsin and allowing up to two missed cleavage sites. Database searching was carried out with a fragment ion mass tolerance of 0.1 Da and a parent ion tolerance of 10 ppm. The iodoacetamide derivative of cysteine was specified as a fixed modification, and oxidation of methionine, N-acetyl-glycosylation of serine and threonine, acetylation of protein N-terminal, and formylation of peptide N-terminal were specified as a variable modification in Mascot. Database search results were validated, and MS/MS spectra were annotated in PeptideShaker 1.16.45 (Vaudel et al., 2015).

### Immunocytochemistry

HeLa cells were grown on coverslips until ∼50% confluence and treated with either 100 μM PUGNAc and 5 mM glucosamine or the vehicle for 1 h at 37°C. Following this, the cells were exposed to 300 mOsmol isotonic (mannitol 100 mM, NaCl 87.5 mM, CaCl_2_ 2.5 mM, MgCl_2_ 2.5 mM, HEPES 10 mM, pH 7.4) or 200 mOsmol hypotonic (NaCl 87.5 mM, CaCl_2_ 2.5 mM, MgCl_2_ 2.5 mM, HEPES 10 mM, pH 7.4) solution for 15 min. Next, cells were fixed in 10 *v*/*v*% PBS-buffered formaldehyde for 30 min at room temperature. To avoid formaldehyde autofluorescence, the coverslips were quenched with 50 mM ammonium chloride for 10 min. The cells were permeabilized with 0.25 *v*/*v*% Triton-X 100 for 10 min. Non-specific sites were blocked with 5% bovine serum albumin (Sigma-Aldrich) in PBS for 30 min, and then, the coverslips were incubated at room temperature with the primary antibodies in 5 *w*/*v*% BSA/PBS for 2 h. The primary antibodies and their dilutions were the following: CTD110.6 (1:200) and rabbit anti-ICln (1:100). After rinsing three times with PBS, samples were incubated with the corresponding secondary antibodies for 1 h in the dark. Finally, coverslips were mounted with VECTASHIELD (Vector Laboratories, Burlingame, CA, United States) mounting medium. Image acquisition was performed with a Zeiss LSM 710 confocal scanning microscope (Carl Zeiss AG, Oberkochen, Germany) equipped with the ZEN software (ver2.3, Carl Zeiss AG, Oberkochen, Germany) and a 63× objective. Alexa Fluor 488 (ICln) and Texas Red (CTD110.6) fluorescence channels were used for image acquisition (excitation 488 and 594 nm, emission 518 and 624 nm, respectively).

### Bioinformatic Analysis

The amino acid sequence of human ICln (NP_001284.1) was analyzed with the YinOYang WWW server (http://www.cbs.dtu.dk/services/YinOYang/), which produces neural network predictions for O-β-GlcNAc attachment sites in eukaryotic protein sequences (Gupta and Brunak, 2002). This server can also use NetPhos, which predicts serine, threonine, or tyrosine phosphorylation sites in eukaryotic proteins, to identify possible phosphorylated sites that, when coinciding with *O*-GlcNAcylation sites, represent “YinOYang” sites (Blom et al., 2004). In addition, the *GlycoMine* (http://glycomine.erc.monash.edu/Lab/GlycoMine/) bioinformatics tool was used to predict O-linked glycosylation (Li et al., 2015).

### Statistical Analysis

All data are expressed as arithmetic means ± standard errors of the mean. For statistical analysis and graphics, GraphPad Prism (version 5.0b for Mac OS, San Diego, CA, United States) and Excel (Microsoft) software were used. Significant differences between mean values were determined by the unpaired Student’s *t*-test or ANOVA with Bonferroni’s or Dunnett’s post-test, as appropriate. Statistically significant differences were determined at ^∗^*p* < 0.05, ^∗∗^*p* < 0.01, ^∗∗∗^*p* < 0.001; (*n*) corresponds to the number of independent measurements.

## Results

### Manipulation of *O*-GlcNAcylation of Cellular Proteins

**FIGURE 1:**
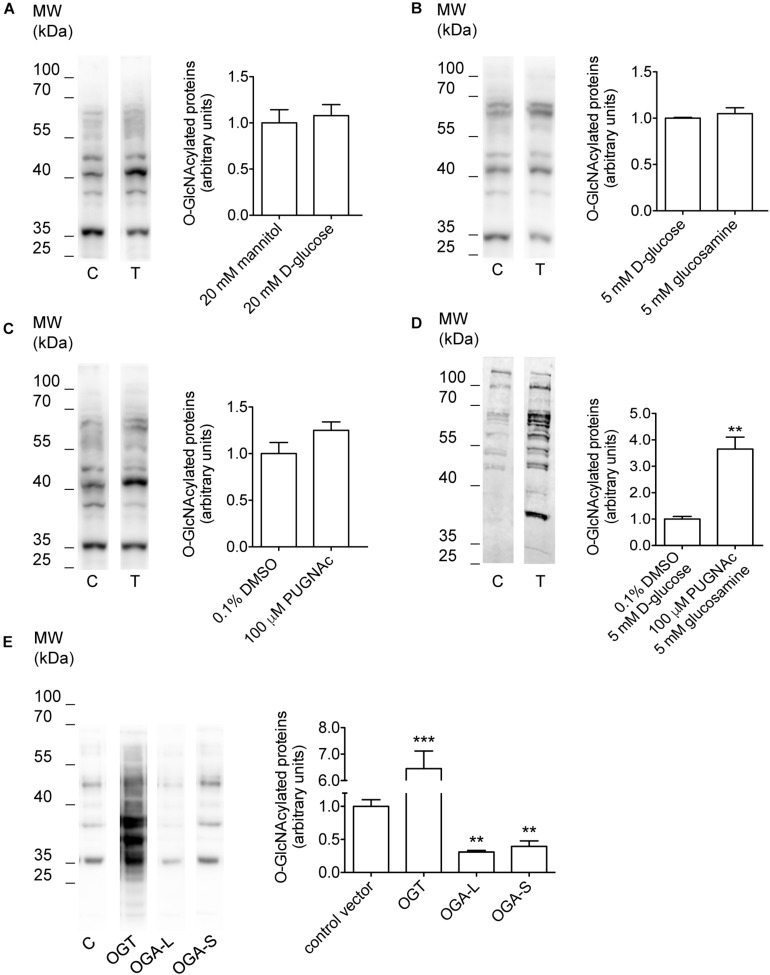
Manipulation of *O*-GlcNAcylation levels of cellular proteins. HEK 293 Phoenix cells were incubated for 1 h in complete medium with **(A)** 20 mM mannitol (control) or D-glucose, **(B)** 5 mM D-glucose (control) or glucosamine, **(C)** 0.1% DMSO (control) or 100 μM PUGNAc, **(D)** 0.1% DMSO plus 5 mM D-glucose (control) or 100 μM PUGNAc plus 5 mM glucosamine, or **(E)** were transfected for 24 h with the pIRES2EGFP vector coding for OGT, OGA-L, or OGA-S or with the pIRES2EGFP-EGFP vector as a control. The expression levels of cellular *O*-GlcNAc-modified proteins determined via western blot were quantified by densitometry and normalized to the housekeeping protein β-actin. *n* = 3, ^∗∗^*p* < 0.01, ^∗∗∗^*p* < 0.001, two-tailed, unpaired Student’s *t*-test or one-way ANOVA with Dunnett’s post-test. (*n*) refers to the number of independent samples. C, control; T, treatment; DMSO, dimethyl sulfoxide; OGT, *O*-GlcNAc-transferase; OGA, *O*-GlcNAcase.

Incubation of cells with *O*-GlcNAc precursors such as D-glucose or glucosamine, pharmacological inhibition of OGA with PUGNAc, as well as overexpression of OGT or OGA have formerly been used to experimentally increase or decrease *O*-GlcNAcylation levels of cellular proteins (Champattanachai et al., 2008; Yuzwa and Vocadlo, 2014; Ferron et al., 2018). Here, incubation of HEK 293 Phoenix cells for 1 h with 20 mM D-glucose, 5 mM glucosamine, or 100 μM PUGNAc failed to significantly increase *O*-GlcNAcylation levels of cellular proteins (Figures 1A–C and Table 1). However, combining 100 μM PUGNAc with 5 mM glucosamine increased *O*-GlcNAcylation up to 3.6-fold of control levels (Figure 1D and Table 1). Transfection of cells with OGT was the most effective maneuver and increased *O*-GlcNAcylation of 6.5-fold compared to cells transfected with a control vector (Figure 1E and Table 1). Conversely, cells expressing either OGA-L or its splicing variant OGA-S showed a significant reduction in *O*-GlcNAcylation of cellular proteins, which was more pronounced with OGA-L (Figure 1E and Table 1). Hence, the catalytic activity of OGA-S was found to be lower than that of OGA-L (Kim et al., 2006). Therefore, only OGA-L was used in further experiments.

**TABLE 1:** Effect of manipulation of *O*-GlcNAcylation of cellular proteins on the current IClswell.

Treatment	% variation in *O*-GlcNAcylation	Effect on IClswell		
		Native cells	Cells transfected with ICln wild type	Cells transfected with ICln Ser67Ala
20 mM D-glucose	+8% (n.s.)	n.s.	n.s.	n.p.
5 mM glucosamine	+5% (n.s.)	n.s.	n.s.	n.p.
100 μM PUGNAc	+25% (n.s.)	n.s.	n.s.	n.p.
100 μM PUGNAc plus 5 mM glucosamine	+265%	n.s.	−55%	n.s.
OGT	+545%	−38%	−58%	−38%
OGA-S	−60%	n.s.	n.p.	n.p.
OGA-L	−69%	n.s.	+158%	n.s.

*The % variation in O-GlcNAcylation was calculated from western blot data shown in Figure 1. The % variation in IClswell intensity was calculated at +100 mV. + and − denote an increase and decrease compared to the corresponding control, respectively. n.s., not significant; n.p., not performed.*

### Increased *O*-GlcNAcylation of Cellular Proteins Inhibits the Endogenous IClswell Current

**FIGURE 2:**
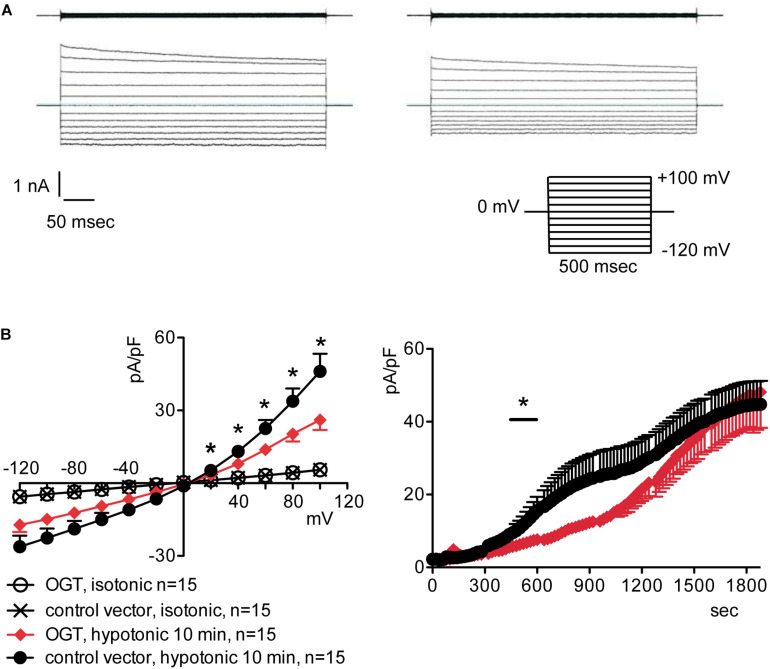
*O*-GlcNAc elevation obtained by OGT overexpression blunts IClswell in native cells. IClswell activation was monitored in HEK 293 Phoenix cells transfected for 24 h with OGT and the transfection marker EGFP as separate proteins or a control vector. Single transfected cells were selected and voltage clamped using the whole-cell patch-clamp technique. **(A)** Original recordings obtained in isotonic (upper panels) and hypotonic (lower panels) extracellular solutions in control (left panels) or OGT-transfected (right panels) cells stimulated with voltage increments of 20 mV from –120 to +100 mV applied from a holding potential of 0 mV (lower right inset). **(B)** Current density-to-voltage relationship determined after a 10-min exposure to the extracellular hypotonic solution (left) and current density-to-time relationship (right) of IClswell in OGT-transfected and control cells. *p < 0.05, unpaired Student’s *t*-test. (*n*) refers to the number of cells. OGT, *O*-GlcNAc-transferase; EGFP, enhanced green fluorescent protein.

To assess the possible influence of *O*-GlcNAc on the endogenous swelling-activated chloride current IClswell, the patch-clamp technique in whole-cell configuration was used in untransfected cells in which the *O*-GlcNAcylation of cellular proteins was experimentally increased or decreased. Cells were initially kept in an extracellular isotonic solution. In these conditions, currents were very small and undistinguishable from leakage currents. Exposure of cells to an extracellular hypotonic solution activated a current with outward rectification and slow time-dependent inactivation at voltages higher than +40 mV. The reversal potential (Erev) of this current was 0 mV, which corresponds to the equilibrium potential of chloride with the experimental solutions used and is therefore indicative of a chloride selective current (Supplementary Figure S1). All of the above-mentioned characteristics represent the biophysical fingerprints of IClswell (Furst et al., 2002a). Pre-incubation of HEK 293 Phoenix cells for 1 h with 20 mM D-glucose, 5 mM glucosamine, or 100 μM PUGNAc failed to increase *O*-GlcNAcylation of cellular proteins (Figures 1A–C) and did not modify the endogenous IClswell current (Supplementary Figures S1–S3 and Table 1). Pre-incubating cells with 100 μM PUGNAc plus 5 mM glucosamine, which was effective in increasing *O*-GlcNAcylation levels in these cells (Figure 1D), also did not significantly modify the endogenous IClswell current (Supplementary Figure S4 and Table 1). In contrast, in cells transfected with OGT, which was twice as effective as 100 μM PUGNAc plus 5 mM glucosamine in increasing *O*-GlcNAcylation levels, IClswell magnitude was significantly reduced and the activation in time was delayed in comparison to control conditions (Figure 2 and Table 1). In cells transfected with OGA-L, IClswell showed a tendency to an increase, which, however, was not statistically significant (Supplementary Figure S5).

### Increased *O*-GlcNAcylation of Cellular Proteins Inhibits the ICln-Induced IClswell Current

**FIGURE 3:**
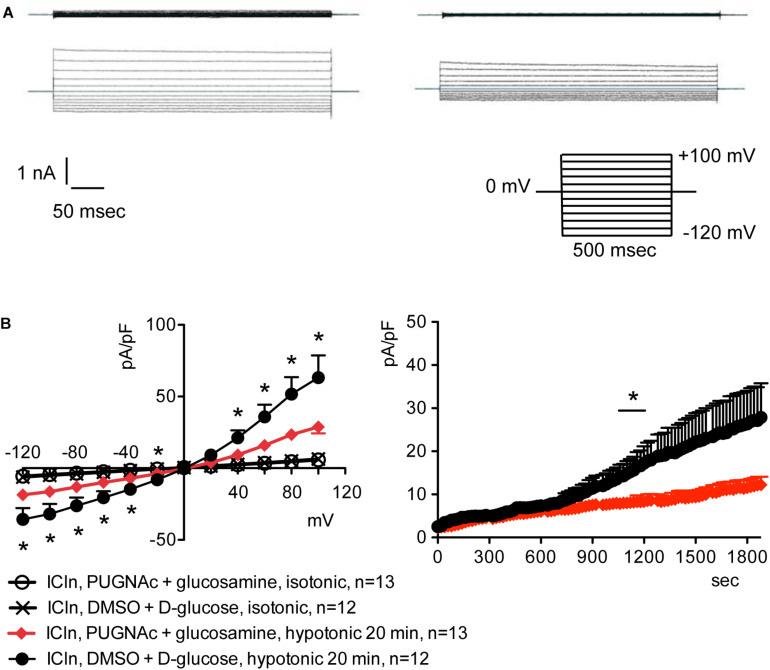
*O*-GlcNAc elevation obtained by pre-incubation of cells with PUGNAc and glucosamine blunts IClswell in ICln-transfected cells. IClswell activation was monitored in HEK 293 Phoenix cells transfected for 24 h with ICln as well as the transfection marker EGFP as separate proteins. Single transfected cells were selected and voltage clamped using the whole-cell patch-clamp technique. **(A)** Original recordings obtained in isotonic (upper panels) and hypotonic (lower panels) extracellular solutions in cells pre-incubated for 1 h with 0.1% DMSO plus 5 mM D-glucose (left panels) or 100 μM PUGNAc plus 5 mM glucosamine (right panels) and stimulated with voltage increments of 20 mV from –120 to +100 mV applied from a holding potential of 0 mV (lower right inset). **(B)** Current density-to-voltage relationship determined after a 20-min exposure to the extracellular hypotonic solution (left) and current density-to-time relationship (right) of IClswell in cells pre-incubated with 100 μM PUGNAc plus 5 mM glucosamine or the vehicle. *p < 0.05, unpaired Student’s *t*-test. (*n*) refers to the number of cells. EGFP, enhanced green fluorescent protein.

**FIGURE 4:**
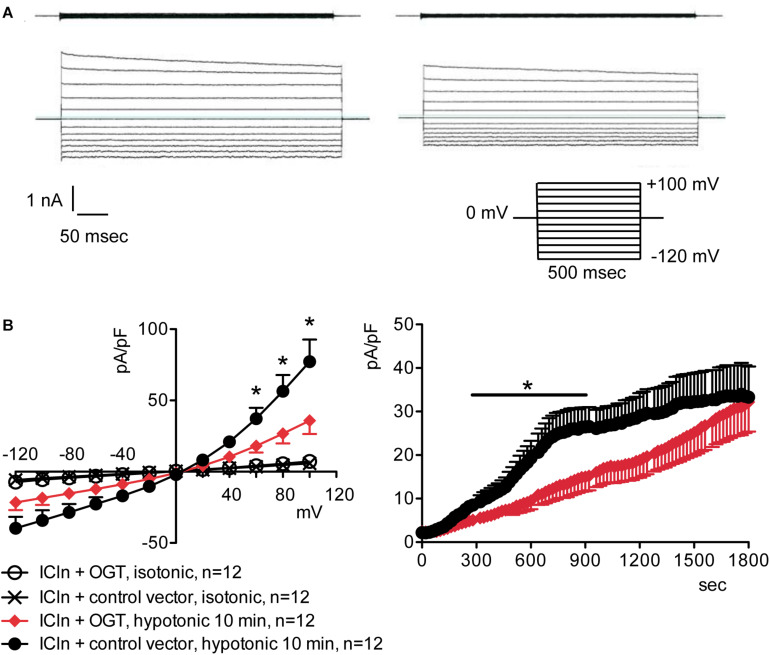
*O*-GlcNAc elevation obtained by OGT overexpression blunts IClswell in ICln-transfected cells. IClswell activation was monitored in HEK 293 Phoenix cells transfected for 24 h with ICln and OGT or ICln and a control vector. Transfection markers were dsRed for ICln and EGFP for OGT. Single cells expressing both transfection markers were selected and voltage clamped using the whole-cell patch-clamp technique. **(A)** Original recordings obtained in isotonic (upper panels) and hypotonic (lower panels) extracellular solutions in control (left panels) or OGT-transfected (right panels) cells stimulated with voltage increments of 20 mV from –120 to +100 mV applied from a holding potential of 0 mV (lower right inset). **(B)** Current density-to-voltage relationship determined after a 10-min exposure to the extracellular hypotonic solution (left) and current density-to-time relationship (right) of IClswell in OGT-transfected and control cells. *p < 0.05, unpaired Student’s *t*-test. (*n*) refers to the number of cells. OGT, *O*-GlcNAc-transferase; EGFP, enhanced green fluorescent protein.

**FIGURE 5:**
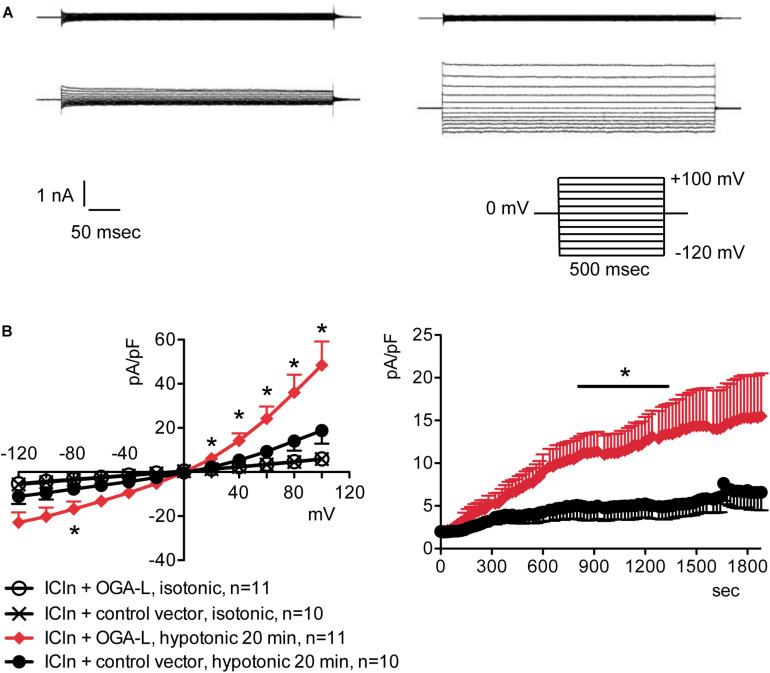
*O*-GlcNAc reduction obtained by OGA-L overexpression upregulates IClswell in ICln-transfected cells. IClswell activation was monitored in HEK 293 Phoenix cells transfected for 24 h with ICln and OGA-L or ICln and a control vector. Transfection markers were dsRed for ICln and EGFP for OGA-L. Single cells expressing both transfection markers were selected and voltage clamped using the whole-cell patch-clamp technique. **(A)** Original recordings obtained in isotonic (upper panels) and hypotonic (lower panels) extracellular solutions in control (left panels) or OGA-L-transfected (right panels) cells stimulated with voltage increments of 20 mV from –120 to +100 mV applied from a holding potential of 0 mV (lower right inset). **(B)** Current density-to-voltage relationship determined after a 20-min exposure to the extracellular hypotonic solution (left) and current density-to-time relationship (right) of IClswell in OGA-L-transfected and control cells. *p < 0.05, unpaired Student’s *t*-test. (*n*) refers to the number of cells. EGFP, enhanced green fluorescent protein; OGA, *O*-GlcNAcase.

Overexpression of the heterologous protein ICln up-regulates IClswell in several cell types (Paulmichl et al., 1992; Hubert et al., 2000; Furst et al., 2002b; Dossena et al., 2011; Schedlbauer et al., 2019) (Supplementary Figure S6). In these cells, the apparent IClswell is the sum of the endogenous IClswell and the ICln-induced IClswell. Similarly to what was observed for the endogenous IClswell current, pre-incubation of cells with 20 mM D-glucose, 5 mM glucosamine, or 100 μM PUGNAc failed to modify IClswell in ICln-transfected cells (Supplementary Figures S7–S9 and Table 1). In contrast, treatment with 100 μM PUGNAc plus 5 mM glucosamine, as well as OGT overexpression, significantly inhibited IClswell (Figures 3, 4 and Table 1). As 5 mM glucosamine plus 100 μM PUGNAc had no effect on the endogenous current, the 55% inhibition of IClswell that was observed in ICln-transfected cells (Table 1) must have arisen from inhibition of the ICln-induced current. Similarly, the OGT-induced IClswell inhibition in ICln-transfected cells (58%) most likely represents the sum of inhibition of (i) the endogenous IClswell (38%, Table 1) and (ii) the ICln-induced current. Conversely, IClswell was significantly up-regulated by OGA-L in ICln-transfected cells (Figure 5 and Table 1).

### Increased *O*-GlcNAcylation of Cellular Proteins Prevents RVD

**FIGURE 6:**
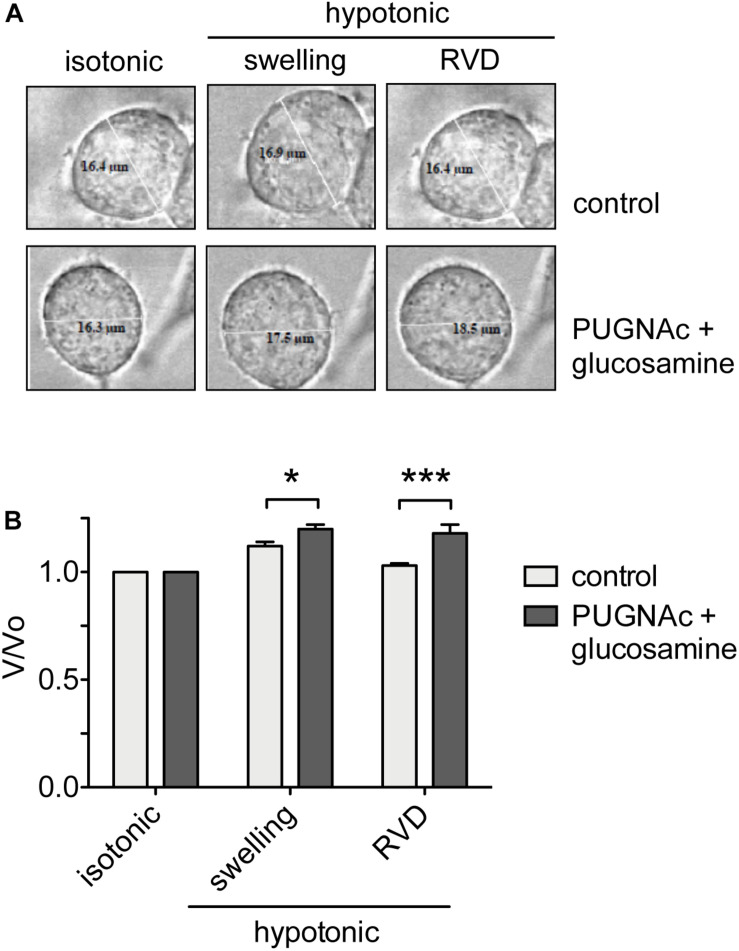
*O*-GlcNAc elevation affects RVD. **(A)** HEK 293 Phoenix cells were incubated for 1 h with 100 μM PUGNAc plus 5 mM glucosamine (*n* = 4) or 0.1% DMSO plus 5 mM glucose as the control (*n* = 5), initially kept in extracellular isotonic solution and then submitted to hypotonic stress. **(B)** *V*/*V*_0_ values determined in extracellular isotonic solution and 30 s (swelling) and 160 s (RVD) after exposure to the hypotonic solution. (*n*) refers to the number of cells. Data are representative of two independent experiments leading to similar results. *p < 0.05, ***p < 0.001, two-way ANOVA with Bonferroni’s post-test. RVD, regulatory volume decrease.

To assess the effects of an increased *O*-GlcNAcylation on RVD, HEK 293 Phoenix cells were treated with 100 μM PUGNAc plus 5 mM glucosamine or 0.1% DMSO plus 5 mM glucose as control. The cell size was monitored before and during an osmotic stress, and cell volume changes were expressed as *V*/*V*_0_. As expected, the hypotonic shock promptly induced cell swelling followed by RVD activation in control cells, which regained their original volume. In contrast, in cells treated with 100 μM PUGNAc and 5 mM glucosamine, cell swelling was more prominent and, importantly, was not followed by an RVD phase (Figure 6).

### The C-Terminus of ICln Is *O*-GlcNAc Modified

**FIGURE 7:**
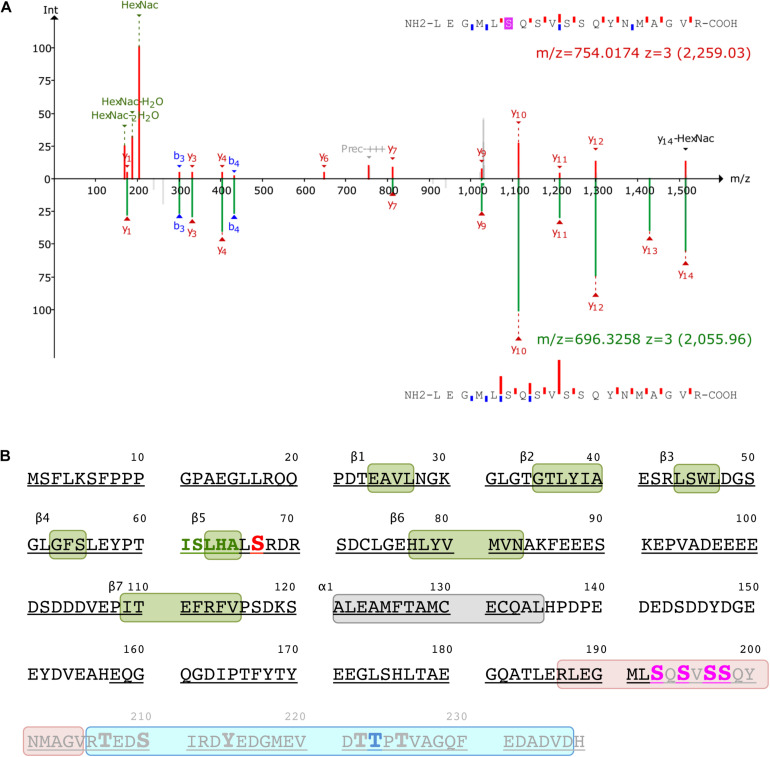
Post-translational modification of ICln. **(A)** Identification of the *O*-GlcNAc site on ICln by LC-MS/MS. Product-ion spectra peptide 187–205 is displayed. Fragmentation products of the *O*-GlcNAc-modified peptide is shown at the top (red) while the unmodified peptide fragments are shown on at the bottom (green) with their corresponding monoisotopic mass *m*/*z* and charge-state (3+). The b- and y-ions are indicated with blue and red, respectively. **(B)** Amino acid sequence of human ICln (NP_001284). Green and gray boxes represent β sheets and α helix secondary structure motifs of the pleckstrin-homology-like domain, respectively. The predicted YinOYang site is in red and the amino acid sequence involved in the interaction with α-integrin is in green. Potential *O*-GlcNAcylation residues within the *O*-GlcNAcylated fragment (187–205, highlighted in rose) are indicated in magenta, and the residue with the highest likelihood of phosphorylation (Thr223) within the phosphorylated fragment (206–236, highlighted in cyan) is indicated in blue. Possible additional phosphorylation sites within this latter fragment are represented as enlarged letters. The amino acids covered by MS are underlined and the fragment missing in the ICln mutant Ser193* is grayed out. MS, mass spectrometry.

Mass spectrometry was used to identify the ICln amino acids that are *O*-GlcNAcylated. After overexpressing ICln in HEK 293 Phoenix cells and treating the cells with 100 μM PUGNAc and 5 mM glucosamine, we successfully identified the ICln protein from immunoprecipitated samples with up to 90% sequence coverage. Out of the 32 individual peptides found, we detected *O*-GlcNAc modification on peptide 187–205. Figure 7A reports the analysis of the charge state 3+ of this peptide. The *O*-GlcNAc-modified peptide has a mass/charge (*m*/*z*) value of 754.014 while its unmodified counterpart has an *m*/*z* value of 696.3258. Supplementary Figure S10 demonstrates the analysis of the same peptide with a charge state of 2+. Unfortunately, no b- or y-ions that carried the *O*-GlcNAc modification could be found; therefore, the exact *O*-GlcNAc site could not be discerned (there are four Ser representing potential *O*-GlcNAcylation sites on this peptide, Figure 7B). However, it is worth noting that both analyses found peaks that corresponded to the *m*/*z* value of GlcNAc residues and probably resulted from a loss of GlcNAc from the parent ion. In addition, we detected phosphorylation at Ser223 on peptide 206–236 (Figure 7B).

### *O*-GlcNAcylation of the C-Terminus of ICln Is Not Involved in Modulating IClswell

Based on the MS results, two mutations were designed in order to prevent ICln *O*-GlcNAcylation, i.e., Ser193^∗^ (nucleotides coding for Ser193 were substituted by a STOP codon) and Thr223Ala (Figure 7B). The first mutation leads to an early termination of translation with consequent protein truncation. The mutant protein lacks all *O*-GlcNAcylation sites, including Ser193. Thr223 is a phosphorylated amino acid with high likelihood of *O*-Glycosylation (Supplementary Figures S11, S12) and therefore a potential YinOYang site. The second mutation converts this amino acid in the non-modifiable Ala. First, the expression of the ICln mutants and their ability of up-regulating IClswell were verified. Subsequently, the effect of an increased *O*-GlcNAcylation of cellular proteins was probed on the mutant ICln-induced current.

**FIGURE 8:**
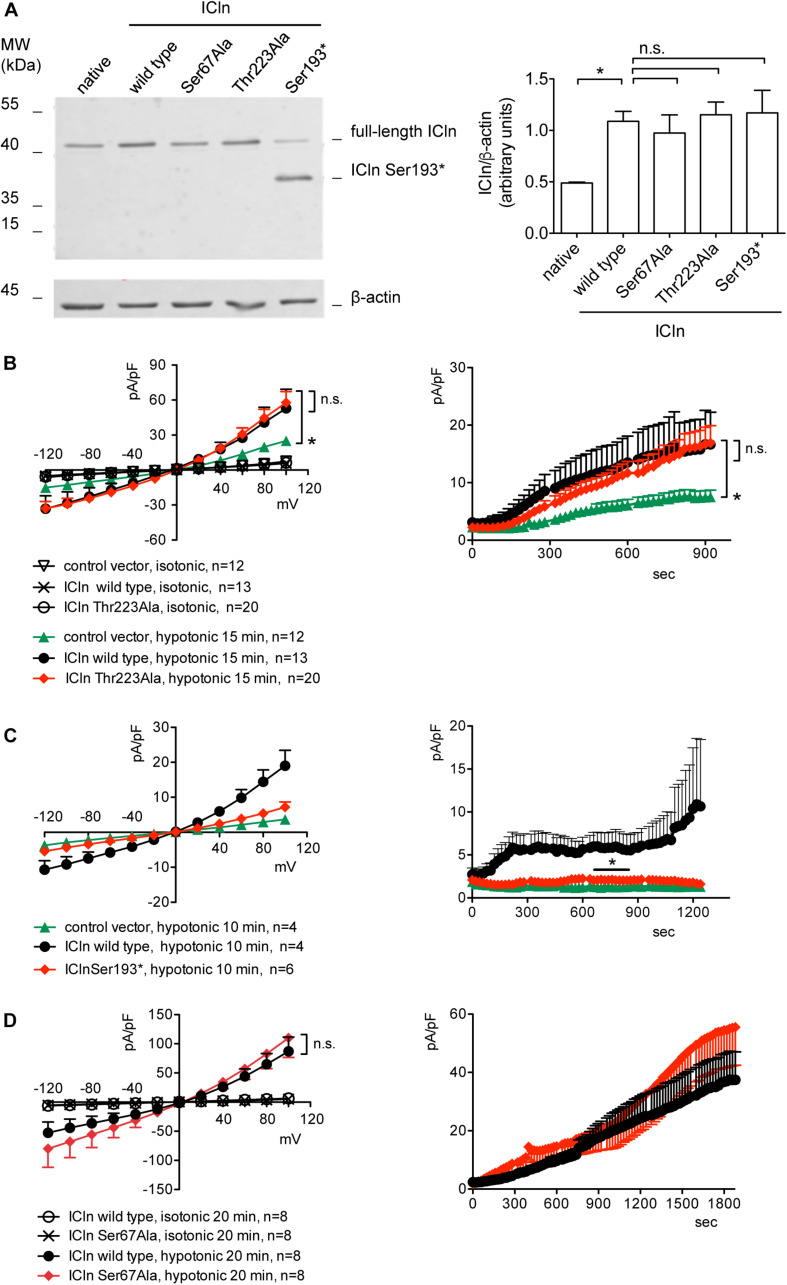
Expression and function of ICln mutants. **(A)** HEK 293 Phoenix cells were transfected for 24 h with the indicated ICln mutants, wild-type ICln, or left untransfected. The expression levels of ICln determined via western blot (left panel) were quantified by densitometry and normalized to the housekeeping protein β-actin (right panel). *n* = 3 independent samples, *p < 0.05, one-way ANOVA with Dunnett’s post-test. **(B–D)** IClswell activation was monitored in HEK 293 Phoenix cells transfected for 24 h with wild-type or mutant ICln or a control vector, as indicated. Single transfected cells were selected based on the fluorescence of the transfection marker EGFP and voltage clamped using the whole-cell patch-clamp technique. The current density-to-voltage relationship (left panels) and the current density-to-time relationship (right panels) determined after exposure to the extracellular hypotonic solution are shown. *p < 0.05, unpaired Student’s *t*-test. n.s., not significant. (*n*) refers to the number of cells. EGFP, enhanced green fluorescent protein.

ICln expression in cells transfected with ICln Thr223Ala and Ser193^∗^ was doubled compared to the expression of the endogenous ICln in untransfected cells and was indistinguishable from that of cells transfected with wild-type ICln (Figure 8A). ICln Thr223Ala was fully functional, i.e., able of up-regulating IClswell to the level of ICln wild type (Figure 8B). In contrast, ICln Ser193^∗^ lost most of its activity, and only a small residual current, which was above the level of the endogenous IClswell, could be measured in transfected cells (Figure 8C).

**FIGURE 9:**
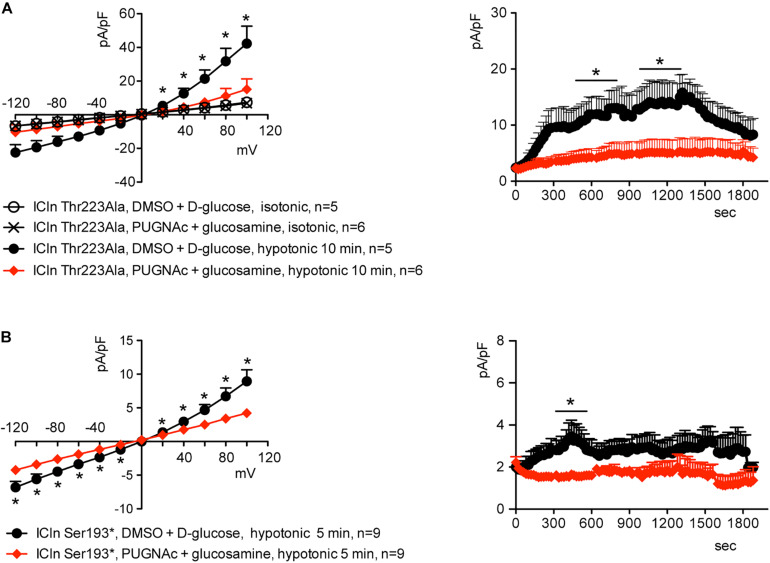
*O*-GlcNAc elevation inhibits IClswell in cells transfected with non-*O*-glycosylatable forms of ICln. IClswell activation was monitored in HEK 293 Phoenix cells transfected for 24 h with **(A)** ICln Thr223Ala or **(B)** ICln Ser193* as well as the transfection marker EGFP as separate proteins and pre-incubated for 1 h with 100 μM PUGNAc plus 5 mM glucosamine or 0.1% DMSO plus 5 mM D-glucose as the control. Single transfected cells were selected and voltage clamped using the whole-cell patch-clamp technique. The current density-to-voltage relationships determined in an extracellular hypotonic solution (left) and the current density-to-time relationships (right) of IClswell in cells pre-incubated with 100 μM PUGNAc plus 5 mM glucosamine or the vehicle are shown. *p < 0.05, unpaired Student’s *t*-test. (*n*) refers to the number of cells. EGFP, enhanced green fluorescent protein.

Similarly to what was observed for the wild-type ICln-induced current, the ICln Thr223Ala-induced IClswell, as well as the residual ICln Ser193^∗^-induced current, were inhibited by increases in the *O*-GlcNAcylation of cellular proteins induced by incubation of cells with PUGNAc and glucosamine (Figure 9). As pre-incubation of cells with PUGNAc plus glucosamine had no effect on the endogenous IClswell (Supplementary Figure S4), the inhibitory effect observed in cells transfected with ICln Thr223Ala or ICln Ser193^∗^ must arise from inhibition of the mutant ICln-induced current. These data clearly show that *O*-GlcNAcylation of ICln on Ser193 or Thr223 is not involved in suppressing IClswell and strongly suggest that the *O*-GlcNAcylation site involved in controlling the ICln-induced current is located upstream of Ser193.

### Ser67 Confers to ICln Sensitivity to *O*-GlcNAcylation

**FIGURE 10:**
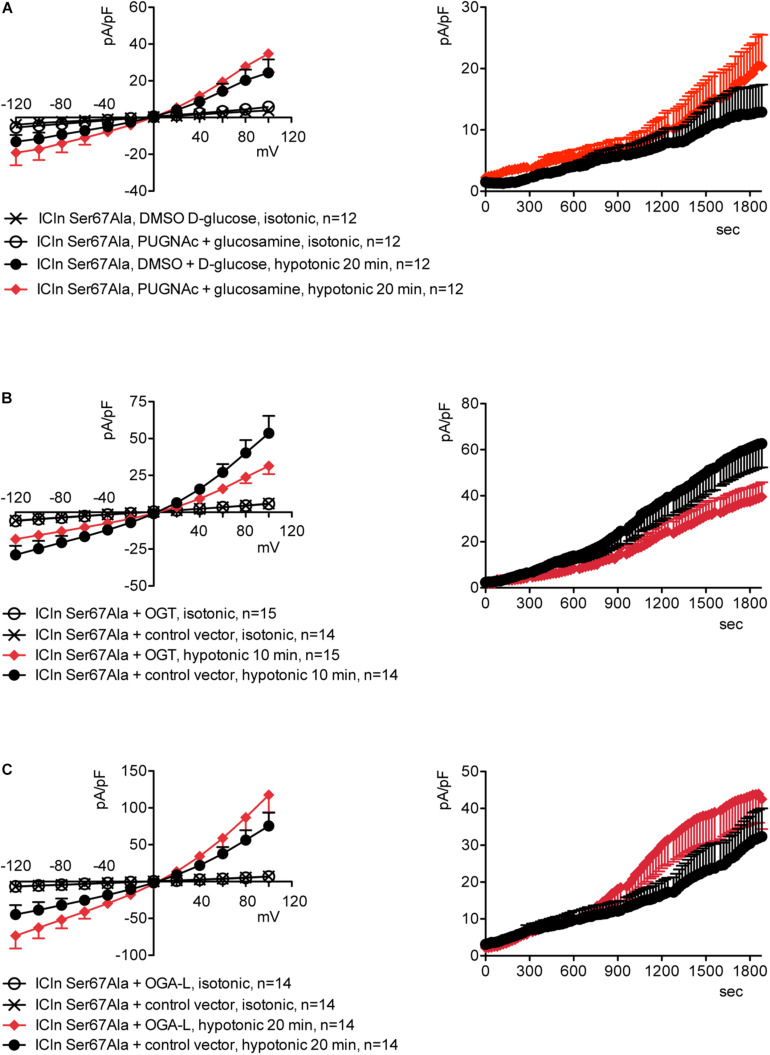
The ICln Ser67Ala-induced IClswell is unresponsive to variations in *O*-glycosylation of cellular proteins. IClswell activation was monitored in HEK 293 Phoenix cells transfected for 24 h with ICln Ser67Ala and the transfection marker dsRed as separate proteins and **(A)** pre-incubated for 1 h with 100 μM PUGNAc plus 5 mM glucosamine or 0.1% DMSO plus 5 mM D-glucose as the control or **(B)** co-transfected with OGT or **(C)** OGA-L and the transfection marker EGFP. The current density-to-voltage relationships determined in an extracellular hypotonic solution (left panels) and the current density-to-time relationships (right panels) of IClswell are shown. (*n*) refers to the number of cells. DMSO, dimethyl sulfoxide; EGFP, enhanced green fluorescent protein. OGT, *O*-GlcNAc transferase; OGA, *O*-GlcNAcase.

Upstream of ICln Ser193, there is a predicted YinOYang site at Ser67 (Supplementary Figure S11). This latter site is of particular interest as it lies in close proximity to the (61)ISLHA(65) motif, which is crucially involved in ICln binding to the intracellular domain of α-integrin and controls ICln function (Schedlbauer et al., 2019). Therefore, we decided to determine whether this site is involved in modulating ICln function following variation of *O*-GlcNAc levels. ICln Ser67Ala is fully functional, i.e., capable of up-regulating IClswell to an extent indistinguishable from that of wild-type ICln (Figure 8D). However, the current induced by this ICln mutant is insensitive to increases or decreases in the *O*-GlcNAcylation of cellular proteins (Figure 10). In particular, OGT overexpression induced a non-significant decrease (38%) in IClswell (Figure 10B and Table 1), which reflected the inhibition of the endogenous current (Figure 2 and Table 1). These experiments strongly suggest that *O*-GlcNAcylation of ICln at Ser67 inhibits IClswell.

### ICln Interacts With OGT

**FIGURE 11:**
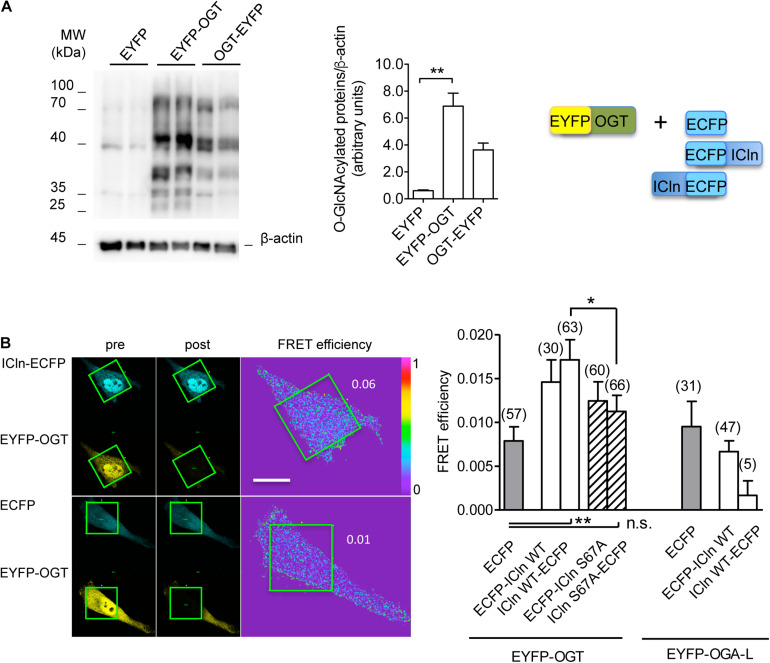
ICln interacts with OGT. HeLa cells were transfected for 48 h with the FRET acceptors EYFP-OGT, OGT-EYFP, or EYFP as a negative control. **(A)** The expression levels of *O*-GlcNAc-modified cellular proteins were determined via western blot (left panel), quantified by densitometry and normalized to the housekeeping protein β-actin (middle panel). *n* = 3 independent samples. The protein pairs selected for FRET experiments are shown (right panel). **(B)** Left panel: single cells transfected with the indicated FRET protein pairs were imaged before (pre) and after (post) photobleaching of the FRET acceptor. Green squares correspond to the bleaching ROI and numbers correspond to the FRET efficiency determined in those ROIs. Scale bar: 10 μm. Right panel: FRET efficiency determined within the bleaching ROIs between the indicated protein pairs. (*n*) refers to the number of cells from four subcultures. **p < 0.01, *p < 0.05, one-way ANOVA with Dunnett’s post-test. n.s., not significant; OGT, *O*-GlcNAc-transferase; FRET, fluorescence resonance energy transfer; EYFP, enhanced yellow fluorescent protein; ROI, region of interest.

Conjugation of *O*-GlcNAc to a protein requires a direct molecular interaction with OGT. To study this phenomenon, FRET between OGT and ICln was measured. This technique requires the transfection of the proteins of interest fused to fluorophores serving as the FRET donor and acceptor. In this study, ECFP and EYFP were used as the FRET donor and acceptor, respectively. EYFP fusion to the N-terminus of OGT did not affect its catalytic activity (Figure 11A) and was therefore used in experiments. EYFP-OGT gave a significant FRET with ICln-ECFP, which was reduced following mutation of Ser67. In contrast, FRET between EYFP-OGA and ICln did not exceed the stochastic FRET of the negative control. These results indicate that, in unstimulated cells, ICln preferentially interacts with OGT rather than OGA, thus suggesting that ICln is constitutively *O*-GlcNAcylated. The ICln–OGT interaction is destabilized by mutation of Ser67, which may therefore represent the ICln *O*-GlcNAcylation site. The relatively small signal-to-noise ration of ICln–OGT FRET indicates that the interaction between the two proteins is dynamic and transient, which is consistent with the characteristics of a reversible interaction between an enzyme and its target.

### *O*-GlcNAcylation of ICln at Ser67 Prevents Its Interaction With α-Integrin

**FIGURE 12:**
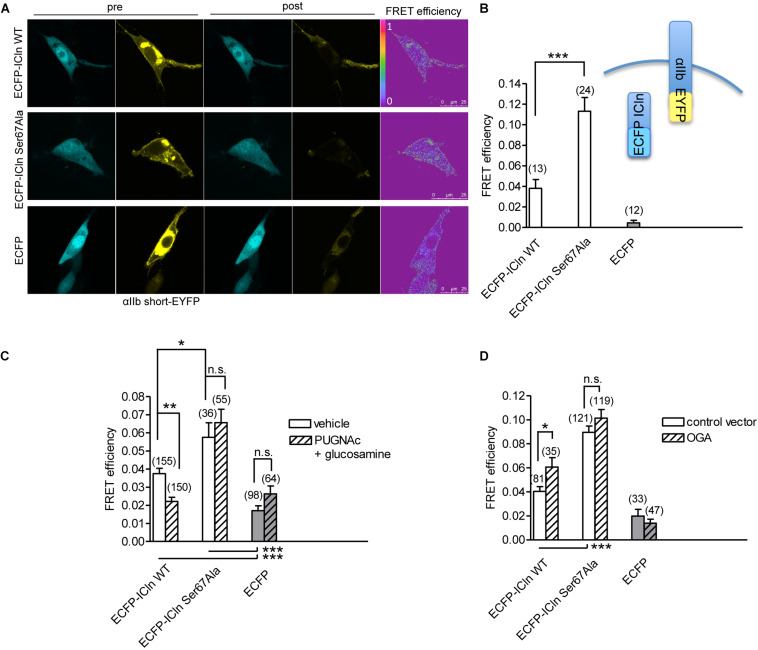
*O*-GlcNAcylation controls the interaction of ICln with α-integrin. **(A)** NIH/3T3 cells were transfected for 48 h with the FRET acceptor αIIbshort-EYFP and ECFP-ICln wild type or Ser67Ala, or with ECFP as a control, and imaged before (pre) and after (post) photobleaching of the FRET acceptor. FRET efficiency was determined in three–four regions of interest (ROIs) of the plasma membrane of each cell. **(B)** The *O*-GlcNAcylation levels were left unmodified or **(C)** increased by incubation of cells with 100 μM PUGNAc plus 5 mM glucosamine for 1 h. The vehicle was 0.1% DMSO plus 5 mM D-glucose. **(D)** The *O*-GlcNAcylation levels were reduced by co-transfection of cells with OGA-L and the transfection marker dsRed as separated proteins. (*n*) refers to the number of ROIs of cells from at least four subcultures. ***p < 0.001, **p < 0.01, *p < 0.05, one-way ANOVA with Dunnett’s post-test. FRET, fluorescence resonance energy transfer; EYFP, enhanced yellow fluorescent protein; ECFP, enhanced cyan fluorescent protein; DMSO, dimethyl sulfoxide.

ICln establishes a direct molecular interaction with the intracellular domain of α-integrin (Larkin et al., 2004, 2009; Daxecker et al., 2008) (Figure 12), and perturbation of this interaction affects IClswell activation (Schedlbauer et al., 2019). Surprisingly, the interaction between the non-glycosylatable/non-phosphorylatable ICln Ser67Ala and αIIb integrin measured by FRET was stronger than the interaction between wild-type ICln and αIIb integrin (Figure 12B). This finding suggests that post-translational modification of Ser67 weakens the binding of ICln to α-integrin. To further substantiate this hypothesis, the interaction between wild type and Ser67Ala ICln was measured following increases or decreases in the *O*-GlcNAcylation of cellular proteins obtained by incubation of cells with PUGNAc and glucosamine or transfection with OGA-L, respectively. In line with the hypothesis, elevation of *O*-GlcNAcylation reduced the FRET between wild-type ICln and α-integrin but, importantly, did not affect the FRET between Ser67Ala ICln and α-integrin (Figure 12C). This confirms that *O*-GlcNAcylation of Ser67 reduces the ICln/integrin interaction and further suggests that a fraction of the ICln protein pool is constitutively *O*-GlcNAcylated. In line with these hypotheses, reduction of *O*-GlcNAcylation increased the FRET between wild-type ICln and α-integrin. Again, Ser67Ala ICln was unresponsive to *O*-GlcNAcylation changes (Figure 12D).

### Hypotonicity Reduces *O*-GlcNAcylation of Cellular Proteins

**FIGURE 13:**
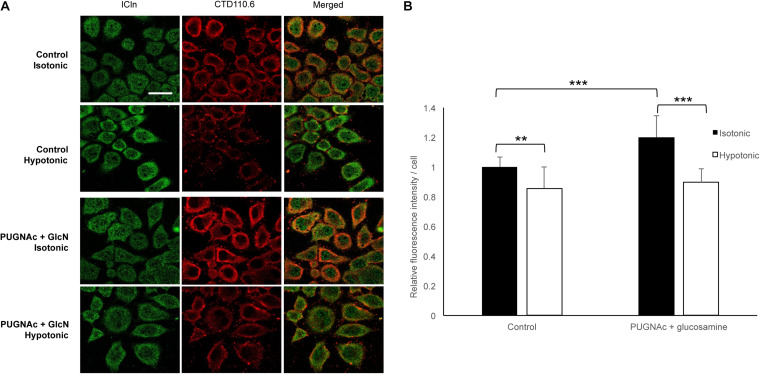
Hypotonicity reduces the cellular *O*-GlcNAcylation levels. Immunofluorescence images of ICln and *O*-GlcNAc proteins in HeLa cells exposed to hypotonic stress. HeLa cells were pre-treated with the vehicle or 100 μM PUGNAc and 5 mM glucosamine (GlcN) and then exposed to normotonic (300 mOsmol) or hypotonic (200 mOsmol) conditions. **(A)** Representative confocal images of cells labeled with anti-ICln (green) and CTD110.6 (anti-*O*-GlcNAc, red) antibodies and corresponding merged images. Scale bar: 20 μM. **(B)** Bar graph shows the average fluorescence intensity of cells normalized to control levels. Each bar represents the average fluorescence of at least 60 cells. Data are shown as means ± SD, **p < 0.01, ***p < 0.001, one-way ANOVA with Dunnett’s post-test.

To assess the dynamic nature of protein *O*-GlcNAc modification upon direct changes during cell volume regulation, we exposed HeLa cells to 200 mOsmol hypotonic challenge. As shown in Figure 13, hypotonicity significantly reduced the fluorescence intensity corresponding to *O*-GlcNAc levels. Interestingly, this effect was also detected when *O*-GlcNAc levels were previously elevated by PUGNAc and glucosamine treatment.

## Discussion

Since the discovery of *O*-GlcNAc modification, more than ∼4,000 protein targets have been identified (Ma and Hart, 2014). A large number of *O*-GlcNAcylated proteins are involved in the regulation of many cellular events, including intracellular signaling, gene transcription, cellular metabolism, and stress responses (Butkinaree et al., 2010; Hart, 2014); therefore, it is not surprising that improper *O*-GlcNAcylation contributes to the development of disease (Hart et al., 2011). Indeed, *O*-GlcNAcylation is associated with an increasing number of pathological states, such as type II diabetes mellitus, diabetic complications (Vaidyanathan and Wells, 2014; Peterson and Hart, 2016), insulin resistance (Wells et al., 2003a), cancer (Stowell et al., 2015), and neurodegeneration (Lagerlof and Hart, 2014; Banerjee et al., 2016; Hart, 2019). Therefore, uncovering the involvement of *O*-GlcNAcylation in a given pathological process and the identification of the respective protein targets could open the way to novel therapeutic strategies.

The alteration of RVD by increases in the *O*-GlcNAcylation of cellular proteins was first reported in Jurkat cells, an immortalized line of human T lymphocytes (Nagy et al., 2010). In this study, exposure of cells for 1 h to 30 mM D-glucose or 5 mM glucosamine was sufficient to significantly alter the levels of *O*-GlcNAcylated proteins as well as the osmotic response of cells to hypotonicity, leading to an increased swelling and delayed RVD. In HEK 293 Phoenix cells, significant increases in *O*-GlcNAc levels required incubation with 100 μM PUGNAc plus 5 mM glucosamine for 1 h (Figure 1D). In agreement with the above-mentioned study, these cells swelled more than control cells when stimulated by hypotonicity, and their RVD was abrogated (Figure 6). These data show that alteration of osmotic resistance by increases in the *O*-GlcNAcylation of cellular proteins is not a feature restricted to Jurkat cells and consists of two components, i.e., increased swelling and inefficient or absent RVD. Jurkat cells seem more sensitive to *O*-GlcNAc precursors than HEK 293 Phoenix cells. This may be linked to differential expression of glucose transporters and/or metabolic enzymes and particularly to the very high expression of the sodium-glucose co-transporter SGLT2 and the glucose transporter GLUT1 in Jurkat cells (Kaji et al., 2018), which may drive efficient glucose intake in these cells.

Inefficient RVD following increases in the *O*-GlcNAcylation of cellular proteins was linked to reduced intracellular water diffusion (Nagy et al., 2010). During RVD, K^+^, Cl^–^, and other intracellular osmolytes are lost, generating an osmotic water efflux that permits cells to reduce their volume (Friedrich et al., 2006). As water follows ions, one may speculate that reduced intracellular water diffusion may arise from impaired ion fluxes. Accordingly, pharmacological inhibition of swelling-activated ion channels leads to increased cell swelling after hypotonic stress and prevents RVD (Furst et al., 2002a), thus mimicking the effect of an increased *O*-GlcNAcylation. Therefore, we hypothesized that *O*-GlcNAcylation may alter RVD by inhibiting ion transport. The finding that the protein ICln, essential in the activation of the swelling-activated Cl^–^ current IClswell, is *O*-GlcNAc-modified (Hahne et al., 2013) further corroborated this hypothesis. As expected, incubation of cells for 1 h with 100 μM PUGNAc plus 5 mM glucosamine significantly inhibited the ICln-induced IClswell (Figure 3), with no effect on the endogenous IClswell (Supplementary Figure S4). Further increase in *O*-GlcNAcylation obtained by OGT overexpression inhibited both the ICln-induced current and the endogenous IClswell current (Figures 2, 4). These findings suggest that the majority of endogenous ICln is *O*-GlcNAcylated in basal conditions and consequently unresponsive to relatively mild increases in *O*-GlcNAcylation, while heterologous ICln is largely unmodified. Experimental conditions that did not significantly increase the *O*-GlcNAcylation levels of cellular proteins as assessed by western blot (Figures 1A–C) failed to suppress the ICln-induced current (Supplementary Figures S7–S9) as well as the endogenous IClswell current (Supplementary Figures S1–S3).

The identification of the *O*-GlcNAcylated proteins and *O*-GlcNAcylated residues of a protein can be challenging. *O*-GlcNAc modifications have low stoichiometry and generally low abundance, and the extremely labile nature of *O*-GlcNAc makes the determination of modification sites by conventional tandem MS (MS/MS) difficult (Wang et al., 2014). Anti-*O*-GlcNAc antibodies did not recognize ICln immunoprecipitated from HEK 293 Phoenix cells, even following elevation of *O*-GlcNAc levels (data not shown). Therefore, computational methods remain an extremely useful tool in the identification of potential *O*-GlcNAcylation sites (Jia and Zuo, 2018). While MS evidenced that ICln is *O*-GlcNAc modified on its C-terminus, this is unlikely involved in the suppression of the ICln-induced current, as mutation or deletion of the C-terminus did not abrogate responsiveness of the ICln-induced current to *O*-GlcNAc elevation (Figure 9). Therefore, we assume that the ICln *O*-GlcNAcylated site involved in the inhibition of the ICln-induced current could not be detected by MS as the *O*-GlcNAc moiety was most likely lost during sample preparation. In line with this hypothesis, mutation of the predicted YinOYang site on Ser67 conferred resistance of the ICln-induced current to *O*-GlcNAc increases or decreases (Figure 10), strongly suggesting that ICln is *O*-GlcNAc modified at Ser67. The non-significant 38% decrease in the ICln Ser67Ala-induced current observed in cells transfected with OGT most likely reflected the effect of *O*-GlcNAc elevation on the endogenous ICln (Figure 10B and Table 1).

The identification of a direct molecular interaction between ICln and OGT confirmed that ICln is *O*-GlcNAc modified. Mutation of Ser67 destabilized the interaction, suggesting that this amino acid comes in close contact with OGT (Figure 11). Substitution of Ser67 with an Ala should have minimal impact on protein conformation, as the expression levels and activity of ICln Ser67Ala are very similar to that of the wild-type protein (Figures 8A,D). Therefore, alterations in interaction with OGT and insensitivity of the ICln Ser67Ala-induced current to *O*-GlcNAc are unlikely linked to a variation in protein folding or stability and most likely reflect an impaired *O*-GlcNAcylation following substitution of Ser67.

Intriguingly, Ser67 lies in close proximity with the amino acid sequence (61)ISLHA(65), which binds the motif KXGFFKR of the intracellular domain of α-integrin as an essential step in IClswell activation (Schedlbauer et al., 2019). This observation prompted us to investigate whether *O*-GlcNAcylation controls the binding of ICln to integrin αIIb. Surprisingly, the FRET efficiency between the non-glycosylatable ICln Ser67Ala and integrin αIIb was approximately thrice that of the wild-type ICln, thus implying a strong interaction (Figure 12A). This finding suggests that *O*-GlcNAcylation prevents the interaction between wild-type ICln and α-integrin and further confirms that wild-type ICln is, at least in part, constitutively *O*-GlcNAcylated. While an increase in the *O*-GlcNAcylation of cellular proteins significantly reduced the direct molecular interaction between wild-type ICln and αIIb, it did not affect ICln Ser67Ala (Figure 12C). In parallel, a decrease in the *O*-GlcNAcylation of cellular proteins significantly increased the interaction between wild-type ICln and αIIb, but did not affect ICln Ser67Ala (Figure 12D). The unresponsiveness of the interaction between ICln Ser67Ala and α-integrin to changes in the *O*-GlcNAcylation levels reflects the insensitivity of the ICln Ser67Ala-induced current to the same conditions (Figure 10). Altogether, these results show that increases in the *O*-GlcNAcylation of cellular proteins affect RVD by inhibiting IClswell; IClswell inhibition may derive from *O*-GlcNAcylation of the protein ICln, with consequent loss of function due to destabilization of the interaction with α-integrin; Ser67 controls the ICln binding to integrin by conferring responsiveness of this interaction to *O*-GlcNAcylation, thus indicating that this or another amino acid located in close proximity most likely represent an *O*-GlcNAcylation site.

An increase in the extracellular osmolarity resulted in an increase in the *O*-GlcNAcylation levels of cellular proteins in mouse embryonic fibroblasts (Zachara et al., 2004), while exposure of HEK293T cells and primary mouse cortical neurons to a hypotonic medium decreased *O*-GlcNAcylation, as a result of a decline in OGT expression (Kommaddi et al., 2011). Accordingly, we found that exposure of HeLa cells to hypotonicity significantly reduced cellular *O*-GlcNAcylation abundance (Figure 13). This event would permit an activation of ICln and, hence, IClswell and RVD, following hypotonic stress. As detailed above, several points of evidence indicate that, in the absence of hypotonic stress, ICln is kept inactive by constitutive *O*-GlcNAcylation. This concept is further supported by the existence of a significant FRET between ICln and OGT in an isotonic environment, while an interaction with OGA could not be detected in the same condition (Figure 11). Altogether, our findings indicate that hypotonicity would lead to IClswell activation via reduction of ICln *O*-GlcNAcylation at Ser67, which would permit its direct binding to α-integrin through the neighboring (61)ISLHA(65) motif.

The amino acid sequence (61)ISLHA(65) appears to be crucial in controlling the interaction between ICln and its molecular partners. As multifunctional protein and connector hub, ICln exerts multiple roles within distinct functional modules by interacting with heterogeneous protein targets (Furst et al., 2006). In addition to its function in the regulation of cellular volume, ICln acts as an assembly chaperone and Smith antigen (Sm) protein mimic and enables the assembly of the 6S core of the small nuclear ribonucleoproteins (snRNP), which are a key component of the spliceosome (Grimm et al., 2013). Within the ring structure of the 6S complex, ICln establishes a direct molecular interaction with SmD1 and SmG. The ICln–SmD1 interaction interface is centered on the antiparallel β-strand pair represented by the SmD1 β4 and the ICln β5. This interaction involves the formation of five backbone–backbone H-bonds involving the residues (61)ISLHA(65) of ICln. A sixth H-bond is formed between the side-chain hydroxyl group of ICln Ser67 and the backbone carbonyl of SmD1 Gln54. Therefore, it is likely that *O*-GlcNAcylation of Ser67 also affects the interaction between ICln and SmD1.

In line with our findings, other studies have shown that RVD and IClswell magnitude were reduced in mouse models of diabetes mellitus, a condition where *O*-GlcNAc is chronically elevated. For example, in white adipocytes isolated from yellow Kuo Kondo (KKA^*y*^) diabetic mice, RVD rate and IClswell magnitude were lower than those measured in adipocytes of normal C57BL/6 or KK mice, a parental strain of KKA^*y*^ mice that do not manifest diabetes until an older age (Inoue et al., 2010). Accordingly, RVD was almost lost and IClswell magnitude was significantly reduced in ventricular myocytes of streptozotocin-induced mice, a well-established model of type 1 diabetes (Yamamoto et al., 2009). Importantly, the RVD rate was heavily reduced also in platelets derived from diabetic patients (Margalit et al., 1995). However, the cellular *O*-GlcNAc levels in these three latter studies were not measured, and the possible link between increased *O*-GlcNAc and impaired RVD was not assessed.

As already mentioned, *O*-GlcNAc modification is involved in the development of insulin resistance and chronic complications of diabetes mellitus (Vaidyanathan and Wells, 2014; Peterson and Hart, 2016), which could in principle be correlated to an impairment of RVD and/or IClswell. Diabetic nephropathy is a clinical syndrome characterized by kidney damage and may result in renal failure (Sun et al., 2013). The cellular and molecular mechanisms leading to diabetic nephropathy are complex and only partially understood. Although the glomerulus has historically been considered as the primary site of diabetic kidney injury, it is becoming increasingly evident that hyperglycemia-induced tubular lesions are prominent components of diabetic nephropathy (Bagby, 2007; Tang and Lai, 2012). The kidney plays the crucial function of controlling the systemic blood pressure and electrolyte balance and is often subjected to osmotic stress during the phenomena of diuresis and anti-diuresis. In addition, in diabetes mellitus, high levels of glucose in the glomerular filtrate lead to an increased reabsorption of glucose and sodium by SGLT2 and SGLT1 in the proximal tubule, along with passive reabsorption of chloride and water (Vallon and Thomson, 2020). It is well known that cells in absorptive epithelia need to efficiently regulate their volume following increases in the intracellular concentration of osmo-active substances, including glucose (van der Wijk et al., 2000). Therefore, although pathological changes like renal fibrosis, mesangial expansion, glomerular hypertrophy, inflammation, and oxidative stress seem to be the main events leading to diabetic nephropathy (Bhattacharjee et al., 2016), we suggest that an impaired or insufficient RVD of kidney tubular cells, which leads to necrotic cell death (Okada et al., 2001, 2019), may contribute to the development of kidney damage in the context of uncontrolled or poorly controlled diabetes mellitus.

Former studies exploring the role of *O*-GlcNAc in diabetic nephropathy mimicked hyperglycemic conditions by exposing various cell types to 20–35 mM D-glucose for 24–48 h (Park et al., 2014; Eleftheriadis et al., 2016, 2020; Gellai et al., 2016). The relatively short incubation time used in the present work (1 h) aimed at disclosing early pathophysiological changes prior overt derangements of cell homeostatic functions occurred. HEK 293 cells, which originate from embryonic kidney, are probably largely undifferentiated but show characteristics of renal epithelial cells and express markers of the proximal tubule, such as SGLT1 (Kaji et al., 2018). HEK 293 cells behavior did not significantly differ from that of HK2 cells—an immortalized proximal tubule epithelial cell line, in an *in vitro* model of diabetic nephropathy (Masola et al., 2011). Other cells used in the present work were chosen as a convenient overexpression system of ectopic proteins. It would be important to confirm the findings presented here, and especially a GlcNAc-induced alteration in RVD, in better models of kidney tubular cells, such as primary human renal proximal tubular epithelial cells.

ICln interacts with multiple partner proteins to participate in multiple cellular functions (Furst et al., 2006). It is tempting to speculate that phosphorylation of Thr223 and/or *O*-GlcNAc modification of the C-terminus of ICln might control its interactome. Further studies are needed to explore this hypothesis.

In conclusion, an increased *O*-GlcNAcylation of cellular proteins leads to impairment of the homeostatic reaction of RVD by suppressing the swelling-activated chloride current IClswell. IClswell suppression is linked to an inhibition of function of the protein ICln subsequent to *O*-GlcNAcylation at Ser67, which leads to destabilization of its direct interaction with the intracellular domain of α-integrin. These results underscore the essential role of *O*-GlcNAc modification in governing basic cell homeostatic functions by controlling protein–protein interactions, and further suggest that reducing ICln *O*-GlcNAcylation may represent a novel strategy in the prevention or treatment of diseases where an RVD derangement might be involved, including chronic complications of diabetes mellitus such as diabetic nephropathy.

## Data Availability Statement

The original contributions presented in the study are included in the article/Supplementary Material, further inquiries can be directed to the corresponding author. The mass spectrometry proteomics data from this study were deposited in the ProteomeXchange Consortium via the jPOST (Okuda et al., 2017) partner repository with the dataset identifier PXD022389 and doi: 10.6019/PXD022389.

## Author Contributions

TN, MP, and SD conceived the study. TN, SD, MP, WP, AS, TJ, AMi, RM, and AMa designed the experiments and interpreted the results. RC, AR, DAC, EB, ZS, TN, and SD performed the experiments and analyzed the data. MP, AMi, AMa, WP, and TJ acquired funding. SD wrote the original draft. WP edited and all authors reviewed and approved the final version of the manuscript.

## Conflict of Interest

The authors declare that the research was conducted in the absence of any commercial or financial relationships that could be construed as a potential conflict of interest.
