# Supplementary material for: O-GlcNAcylation Suppresses the Ion Current IClswell by Preventing the Binding of the Protein ICln to α-Integrin
Source: Front Cell Dev Biol. 2020 Nov 19;8:607080. doi: 10.3389/fcell.2020.607080 (PMC7717961; doi:10.3389/fcell.2020.607080)
Supplement: Supplementary file 1 [file Data_Sheet_1.docx]

Supplementary Material

## Supplementary Figures

**Supplementary Figure 1.** Treatment of cells with D-glucose does not affect IClswell. IClswell activation was monitored in HEK 293 Phoenix cells pre-incubated for 1 hour with 20 mM D-glucose or 20 mM mannitol as the control. Single cells were selected and voltage clamped using the whole-cell patch clamp technique. (**A**) Original recordings obtained in isotonic (upper panels) and hypotonic (lower panels) extracellular solutions in control cells (left panels) or cells treated with D-glucose (right panels) stimulated with voltage increments of 20 mV from -120 to +100 mV applied from a holding potential of 0 mV (lower right inset). (**B**) Current density-to-voltage relationship determined after a 20 minutes exposure to the extracellular hypotonic solution (left) and current density-to-time relationship (right) of IClswell in cells treated with D-glucose and in control cells. (n) refers to the number of cells.

**Supplementary Figure 2.** Treatment of cells with glucosamine does not affect IClswell. IClswell activation was monitored in HEK 293 Phoenix cells pre-incubated for 1 hour with 5 mM glucosamine or 5 mM D-glucose as the control. Single cells were selected and voltage clamped using the whole-cell patch clamp technique. (**A**) Original recordings obtained in isotonic (upper panels) and hypotonic (lower panels) extracellular solutions in control cells (left panels) or cells treated with glucosamine (right panels) stimulated with voltage increments of 20 mV from -120 to +100 mV applied from a holding potential of 0 mV (lower right inset). (**B**) Current density-to-voltage relationship determined after a 20 minutes exposure to the extracellular hypotonic solution (left) and current density-to-time relationship (right) of IClswell in cells treated with glucosamine and in control cells. (n) refers to the number of cells.

**Supplementary Figure 3.** Treatment of cells with PUGNAc does not affect IClswell. IClswell activation was monitored in HEK 293 Phoenix cells pre-incubated for 1 hour with 100 μM PUGNAc or 0.1% DMSO as the control. Single cells were selected and voltage clamped using the whole-cell patch clamp technique. (**A**) Original recordings obtained in isotonic (upper panels) and hypotonic (lower panels) extracellular solutions in control cells (left panels) or cells treated with PUGNAc (right panels) stimulated with voltage increments of 20 mV from -120 to +100 mV applied from a holding potential of 0 mV (lower right inset). (**B**) Current density-to-voltage relationship determined after a 20 minutes exposure to the extracellular hypotonic solution (left) and current density-to-time relationship (right) of IClswell in cells treated with PUGNAc or the vehicle. (n) refers to the number of cells.

**Supplementary Figure 4.** O-GlcNAc elevation obtained by pre-incubation with PUGNAc and glucosamine does not affect IClswell in native cells. HEK 293 Phoenix cells were pre-incubated for 1 hour with 100 μM PUGNAc plus 5 mM glucosamine or 0.1% DMSO plus 5 mM D-glucose as the control. Single cells were selected and voltage clamped using the whole-cell patch clamp technique. (**A**) Original recordings obtained in isotonic (upper panels) and hypotonic (lower panels) extracellular solutions in control cells (right panels) and cells treated with PUGNAc and glucosamine (left panels), stimulated with voltage increments of 20 mV from -120 to +100 mV applied from a holding potential of 0 mV (lower right inset). (**B**) Current density-to-voltage relationship determined after a 20 minutes exposure to the extracellular hypotonic solution (left) and current density-to-time relationship (right) of IClswell in cells treated with PUGNAc plus glucosamine and control cells. (n) refers to the number of cells.

**Supplementary Figure 5.** O-GlcNAc elevation obtained by OGA-L overexpression does not significantly modify IClswell in native cells. IClswell activation was monitored in HEK 293 Phoenix cells transfected for 24 hours with OGA-L and the transfection marker EGFP or a control vector. Single transfected cells were selected and voltage clamped using the whole-cell patch clamp technique. (**A**) Original recordings obtained in isotonic (upper panels) and hypotonic (lower panels) extracellular solutions in control (left panels) or OGA-L transfected (right panels) cells stimulated with voltage increments of 20 mV from -120 to +100 mV applied from a holding potential of 0 mV (lower right inset). (**B**) Current density-to-voltage relationship determined after a 20 minutes exposure to the extracellular hypotonic solution (left) and current density-to-time relationship (right) of IClswell in cells transfected with OGA-L and control cells. (n) refers to the number of cells.

**Supplementary Figure 6.** ICln upregulates IClswell. IClswell activation was monitored in HEK 293 Phoenix cells transfected for 24 hours with ICln and the transfection marker EGFP or a control vector. Single transfected cells were selected and voltage clamped using the whole-cell patch clamp technique. (**A**) Original recordings obtained in isotonic (upper panels) and hypotonic (lower panels) extracellular solutions in control (left panels) or ICln transfected (right panels) cells stimulated with voltage increments of 20 mV from -120 to +100 mV applied from a holding potential of 0 mV (lower right inset). (**B**) Current density-to-voltage relationship determined after a 20 minutes exposure to the extracellular hypotonic solution (left) and current density-to-time relationship (right) of IClswell in ICln-transfected and control cells. *p<0.05, unpaired Student’s t-test. (n) refers to the number of cells.

**Supplementary Figure 7.** Treatment with D-glucose does not affect IClswell in ICln-transfected cells. IClswell activation was monitored in HEK 293 Phoenix cells transfected for 24 hours with ICln and the transfection marker EGFP as separate proteins. Single transfected cells were selected and voltage clamped using the whole-cell patch clamp technique. (**A**) Original recordings obtained in isotonic (upper panels) and hypotonic (lower panels) extracellular solutions in cells pre-incubated for 1 hour with 20 mM mannitol as the control (left panels) or 20 mM D-glucose (right panels) and stimulated with voltage increments of 20 mV from -120 to +100 mV applied from a holding potential of 0 mV (lower right inset). (**B**) Current density-to-voltage relationship determined after a 20 minutes exposure to the extracellular hypotonic solution (left) and current density-to-time relationship (right) of IClswell in cells treated with D-glucose and control cells. (n) refers to the number of cells.

**Supplementary Figure 8.** Treatment with glucosamine does not affect IClswell in ICln-transfected cells. IClswell activation was monitored in HEK 293 Phoenix cells transfected for 24 hours with ICln and the transfection marker EGFP as separate proteins. Single transfected cells were selected and voltage clamped using the whole-cell patch clamp technique. (**A**) Original recordings obtained in isotonic (upper panels) and hypotonic (lower panels) extracellular solutions in cells pre-incubated for 1 hour with 5 mM D-glucose as the control (left panels) or 5 mM glucosamine (right panels) and stimulated with voltage increments of 20 mV from -120 to +100 mV applied from a holding potential of 0 mV (lower right inset). (**B**) Current density-to-voltage relationship determined after a 20 minutes exposure to the extracellular hypotonic solution (left) and current density-to-time relationship (right) of IClswell in cells treated with glucosamine and control cells. (n) refers to the number of cells.

**Supplementary Figure 9.** Treatment with PUGNAc does not affect IClswell in ICln-transfected cells. IClswell activation was monitored in HEK 293 Phoenix cells transfected for 24 hours with ICln and the transfection marker EGFP as separate proteins. Single transfected cells were selected and voltage clamped using the whole-cell patch clamp technique. (**A**) Original recordings obtained in isotonic (upper panels) and hypotonic (lower panels) extracellular solutions in control cells pre-incubated for 1 hour with 0.1% DMSO as the vehicle (left panels) or 100 μM PUGNAc (right panels) and stimulated with voltage increments of 20 mV from -120 to +100 mV applied from a holding potential of 0 mV (lower right inset). (**B**) Current density-to-voltage relationship determined after a 20 minutes exposure to the extracellular hypotonic solution (left) and current density-to-time relationship (right) of IClswell in cells treated with PUGNAc or the vehicle. (n) refers to the number of cells.

**Supplementary Figure 10.** Identification of the O-GlcNAc site on ICln by LC-MS/MS. Product-ion spectra peptide 187-205 is displayed. Fragmentation products of the O-GlcNAc modified peptide is shown at the top (red) while the unmodified peptide fragments are shown at the bottom (green) with their corresponding monoisotopic mass m/z and charge-state (2+). The b- and y-ions are indicated with blue and red, respectively.


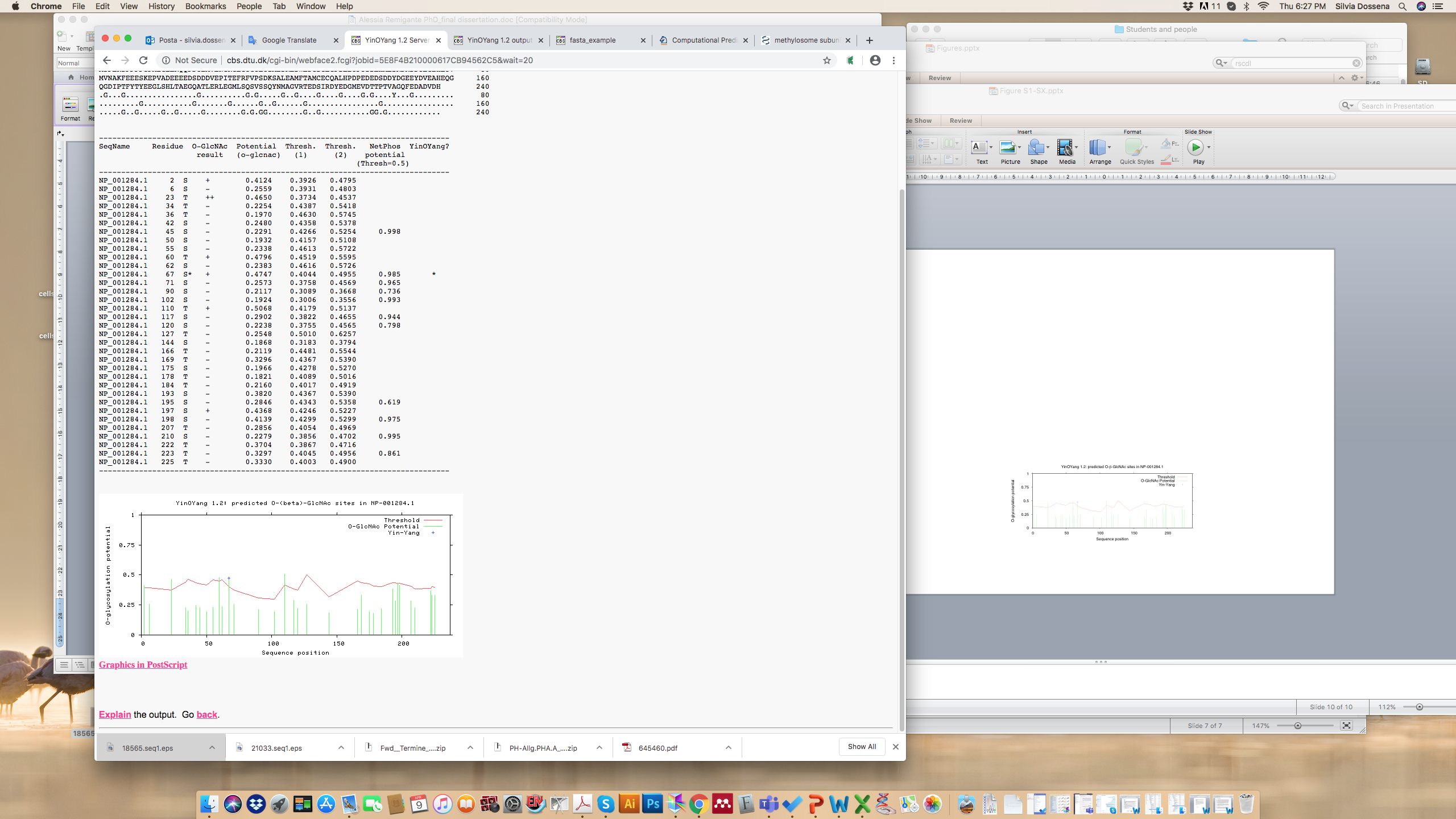


A

B

**Supplementary Figure 11.** Potential O-GlcNacylation and phosphorylation sites on the amino acid sequence of human ICln (NP_001284) according to the Yin-O-Yang WWW algorithm (see Methods). (**A**) Sites marked with a -, + or ++ do not reach the O-GlcNAc threshold, reach the less stringent O-GlcNAc threshold 1 or the more stringent O-GlcNAc threshold 2, respectively. Ser/Thr residues which are predicted to be O-GlcNAcylated as well as phosphorylated are marked by an asterisk (*). (**B**) O-GlcNAc potential and its threshold and Yin-O-Yang site(s) represented across the length of the ICln amino acid sequence from the N-terminal to the C-terminal.


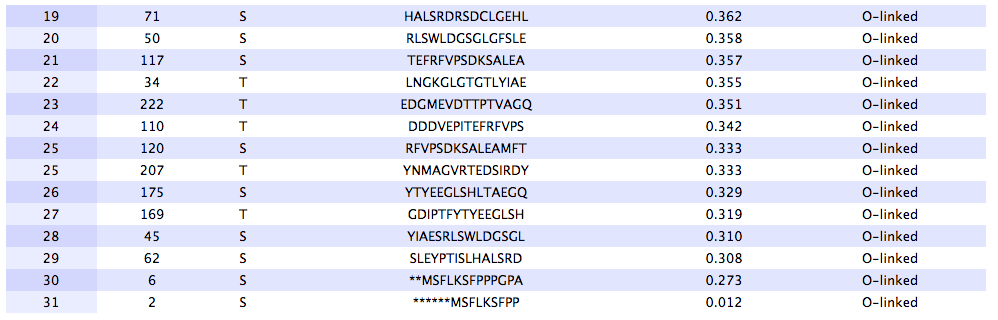

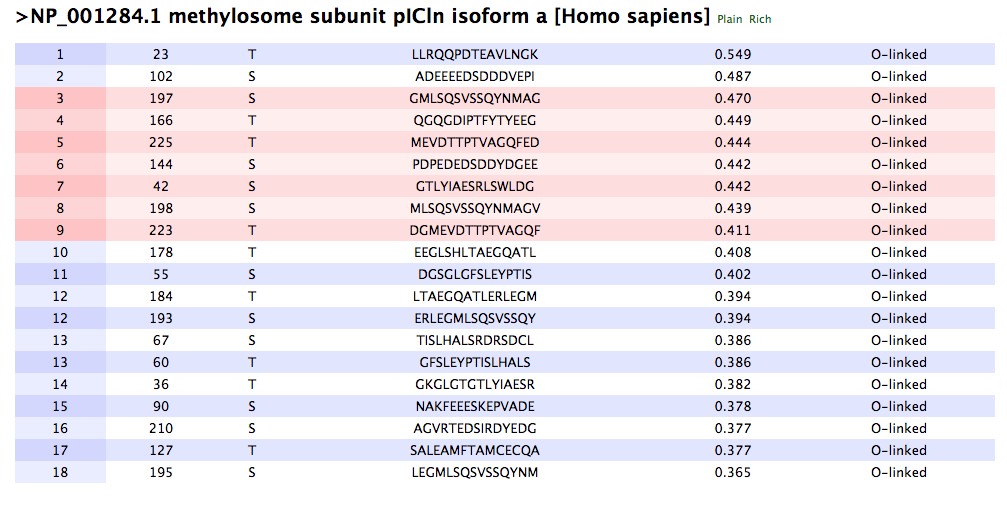


**Rank Position Site Motif Score Type**

**Supplementary Figure 12.** Potential O-GlcNacylation sites on the amino acid sequence of human ICln (NP_001284) according to the GlycoMine algorithm (see Methods).
